# Standards for Waveform Metrology Based on Digital Techniques

**DOI:** 10.6028/jres.095.034

**Published:** 1990

**Authors:** Barry A. Bell

**Affiliations:** National Institute of Standards and Technology, Gaithersburg, MD 20899

**Keywords:** calibration, data conversion, digital synthesis, harmonic distortion, phase angle, waveform sampling

## Abstract

Over the last decade the use of digital synthesis and sampling techniques for generating and measuring electrical waveforms has increased dramatically with the availability of improved digital-to-analog (D/A) and analog-to-digital (A/D) converters and related devices. With this evolution has come the need for physical laboratory standards and test methods to support the performance specifications of digital devices and the instruments in which they are used. This article describes the research and development at NIST of several laboratory standards and test systems that utilize “digital technology” for characterizing data converters and for implementing various waveform synthesis and sampling instruments.

## 1. Introduction

In the early 1970s the NAS/NRC Evaluation Panel for the Electricity Division of the National Bureau of Standards (NBS) made recommendations that the Electrical Instruments Section begin to address the metrology problems associated with precision electrical/electronic instruments and test equipment where dynamic performance considerations were becoming paramount. In September 1974, a workshop was held at the NBS Gaithersburg facility to identify critical metrology needs associated with modern electronic instrumentation. The discussion topics, session notes, feedback reports, and conclusions of the workshop are well documented in an NBS Technical Note [1]. A general theme running through the summaries of the workshop discussions was the need for NBS to provide improved metrology support for the digital technology being incorporated in electrical/electronic devices, test equipment, and systems, and to address dynamic as well as static test conditions.

A number of specific project activities recommended by the Panel and the 1974 Workshop were undertaken by what has since been renamed the Electronic Instrumentation and Metrology (EIM) Group [2]. This article is a synopsis of the description of some of the new physical laboratory standards, together with their operating principles and performance, that have come out of this research. The work to be described covers three general areas in the following order: data converter characterization, generating reference waveforms digitally, and measuring waveform parameters using digital sampling. These three areas are summarized briefly below.

### 1.1 Data Converter Characterization

Since a critical part of “digital” measurement technology involves the basic principles associated with converting analog signals to digital form and vice versa, an initial project was started for providing a basis by which to test precision data converters (both digital-to-analog (D/A) and analog-to-digital (A/D) converters, often designated as DACs and ADCs). A precision 20-bit D/A converter (DAC 20) was developed during the late 1970s to serve as the reference against which 12- to 18-bit converters could be compared [3]. A static test set was developed for providing these comparison measurements automatically [4]. A test set was also developed for measuring the dynamic performance of ADCs with up to 16 bits of resolution [5]. With the ability to quantify the linearity, offset, and gain errors of precision converters (both statically and dynamically), the limitations in the performance of these devices can be determined when used for synthesizing and sampling analog waveforms.

### 1.2 Generating Reference Waveforms Digitally

Concurrent with the development of DAC 20 was an effort to develop a precision audio-frequency phase angle standard that utilized 16-bit DACs to generate two output waveforms whose relative phase difference could be established digitally rather than with analog bridge methods [6,7]. By making use of 18-bit computations and 16-bit DACs, both the amplitude and phase of the two output waveforms from a dual-channel generator were digitally controlled in order to provide a low-frequency ac power source with amplitude and phase stability of 20 ppm and 1 μrad, respectively, over a 100-s measurement period [8]. By using two DACs to prevent “glitches” in the stepped output waveform, a calculable rms ac voltage source has recently been developed. It can be used as a transportable ac reference standard with uncertainties at the 7-V rms level typically less than ±10 ppm from 30 Hz to 15 kHz [9]^1^. These physical standards are being used in several different automatic test systems at NIST to provide calibration services for phase angle meters, wattmeters and watthour meters (also var and varhour meters), and ac digital voltmeters.

### 1.3 Measuring Waveform Parameters Using Digital Sampling

The characterization of sample and hold (S/H) amplifiers and ADCs for sampling and digitizing waveforms was also part of the EIM Group’s development efforts during the 1970s [10,11]. An early application of precision synchronous waveform sampling was the development of a digital wattmeter that used 15 bit successive approximation ADCs, phase locked to the input signals, to provide power measurements good to an uncertainty of ±0.02% from dc to 2 kHz [12]. Using a 12-bit resolution data acquisition module (S/H, instrumentation amplifier, and ADC), together with an internal microcomputer, a low-frequency sampling voltmeter was developed that acquired ac voltage input signals below 10 Hz good to an uncertainty of ±0.1% with a total autoranging, settling, and measurement time of only two signal periods [13]. In the early 1980s the principles of asynchronous sampling of corresponding voltage and current signals were studied, and a wideband sampling wattmeter was developed, capable of measuring audio frequency power from 1 Hz to 10 kHz (with distortion harmonics up to 100 kHz) with an uncertainty of less than ±0.1% [14]. More recently, an equivalent-time sampling and digitizing system, based on a sampling voltage tracker (SVT) circuit, has been developed that has been shown to be capable of making state-of-the-art measurements of signals with frequencies up to 200 MHz [15]. These sampling instruments are also being used in both stand-alone and automatic test sets for calibrating wideband power meters, low-frequency voltmeters, and step/arbitrary waveform generators.

## 2. Data Converter Characterization

A number of special methods have been developed for testing various performance parameters of data converters, and commercial, general-purpose test sets for these devices are now available. However, these methods and systems typically are not capable of measuring the integral linearity, differential linearity, gain, and offset errors of 12- to 18-bit converters good to fractions of a least significant bit (LSB). (An LSB represents the resolution of a converter and is defined as (full scale range)/2*_n_* where *n* is the number of bits of the converter.) For quantifying the accuracy errors of 12-to 18-bit data converters, test methods have been developed and test sets are available at NIST. A NIST calibration service for data converters is offered, as described in [16].

### 2.1 Measuring Static Transfer Characteristics

**Figure 1:**
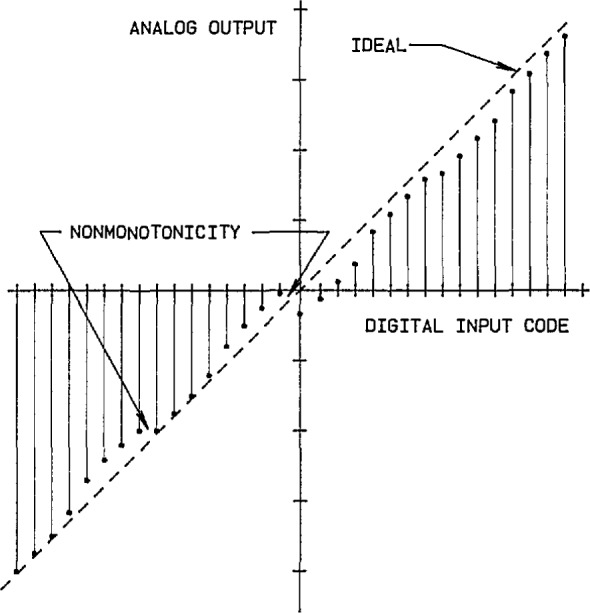
The transfer characteristics for an ideal and an actual DAC.

**Figure 2:**
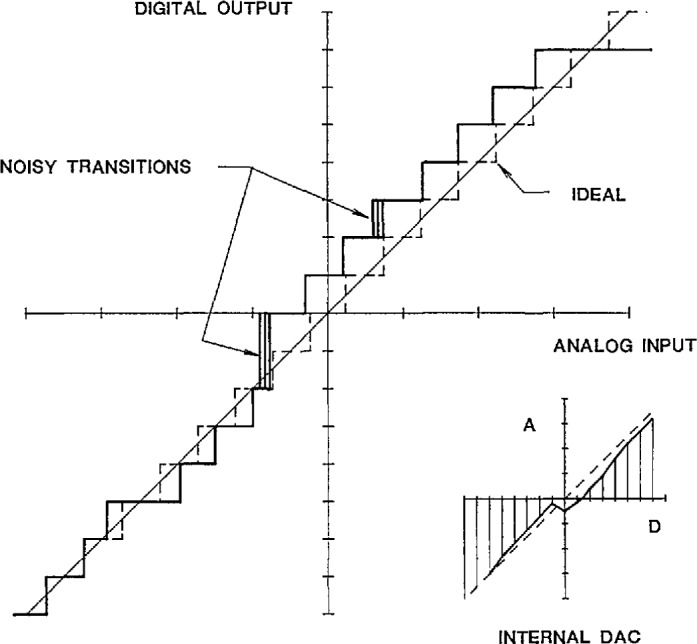
The transfer characteristics for an ideal and an actual ADC.

The primary property of concern when characterizing precision data converters is the relationship between the input and output variables under both static and dynamic conditions. The transfer characteristic for a DAC is shown in figure 1, where the straight dashed line indicates the ideal relationship between the digital input codes and the corresponding analog output levels. Due to offset voltages/currents, mismatches in the divider circuit (typically an R-2R ladder network), and other sources of nonlinearities, the actual characteristic deviates from the ideal. When an increase in the digital input code does not produce a corresponding increase in the analog output level, the behavior is described as nonmonotonic. Similarly, the transfer characteristic for an ADC is shown in figure 2, where the perfect “staircase” relationship between the analog input levels and the corresponding digital output codes represents the ideal. Due to noise from the logic circuits, speed limitations of the analog comparator, errors of the internal reference DAC, etc., the actual ADC characteristic has the non-uniform steps and non-ideal edge transitions indicated in figure 2. Finding and quantifying these small deviations can be important in critical applications of precision data converters.

**Figure 3:**
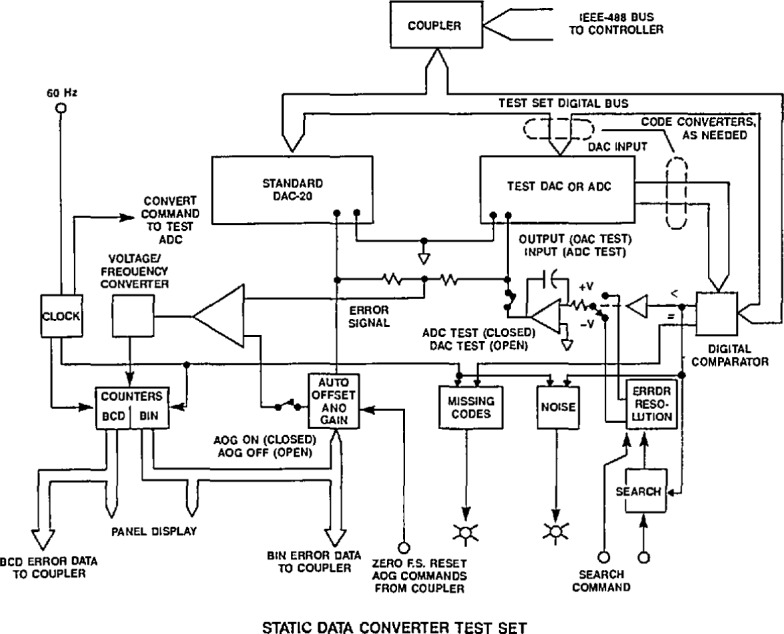
A simplified diagram of the NIST static data converter test set.

Figure 3 shows a simplified diagram of the NIST test set for measuring the essential parameters of the static transfer characteristic of precision data converters. The basic operation involves a comparison of outputs of the test DAC or ADC with the corresponding levels from the NIST DAC 20. This multirange, 20-bit+sign, programmable voltage source exhibits less than 1 ppm linearity error and incorporates a self calibration feature [3]. The design is based on an R-2R ladder network in the current steering mode, using miniature latching relays (to minimize thermal effects) with mercury-wetted contacts for the switches. Ten buffered, temperature-controlled, unsaturated standard cells (providing direct linkage to the legal volt) comprise the voltage reference.

For testing DACs, the DAC 20 standard and the test unit are given the same digital input code from the system controller via an IEEE-488 bus and coupler, with code conversion as needed. The difference in their analog output levels (error signal) is then amplified, digitized with a voltage to frequency (V/F) converter, and averaged over a 60-Hz period (to reduce power frequency noise). The error data (both binary and BCD coded) are displayed on front panel readouts and returned to the controller for logging and further analysis. By sequencing through all 2*^n^* code states (or only 1024, corresponding to the ten most significant bits), the complete DAC static transfer characteristic can be determined with a full scale output resolution of 1 part in 2^20^.

Testing an ADC is more complicated in that each digital output code from the test unit corresponds to a small *continuous range* of analog input values, as illustrated in figure 2. A complete determination of the ADC transfer characteristic requires locating and measuring each of the voltage levels corresponding to the transitions between digital codes. Transition level location in the NIST static data converter test set is accomplished by placing the test ADC in a feedback loop that controls the input voltage to the ADC, as shown in figure 3. The upper digital code of the transition level of interest is input over the IEEE-488 bus from the controller to a digital comparator whose other digital input comes from the ADC output (with code conversion, as needed). The digital comparator’s “less than” output sets the polarity of a programmable voltage (+V, −V), which is switched to the input of an integrating amplifier. Thus, the output voltage from the integrator, switched to the input of the test ADC, ramps up (or down) until the upper transition level code is output from the ADC, whereupon the less than output from the digital comparator changes state, causing the slope of the integrator’s output voltage to reverse. The ADC then outputs the lower transition level code so that the process continues at a preset clock rate (normally 10 kHz), generating a small (≪1 LSB) peak-to-peak triangle waveform at the ADC input with an average value very near the transition level. Once locked onto a transition level, that voltage is then measured by the same comparison circuitry described above for DAC testing. The error data are collected by sequencing through 1024 (or, all 2*^n^* if desired) code states in order to determine the complete ADC static transfer characteristic. Since bits less significant than the 10th generally contribute insignificant errors, typically only 2^10^=1024 codes are tested, corresponding to the 10 most significant bits.

#### 2.1.1 Analyzing Static Linearity Errors

At each of the 1024 codes the test set automatically measures the errors, defined as the difference between the output (input) of the DAC (ADC) under test and the output of reference DAC 20 (calibrated to have negligible linearity errors). These measured errors are then numerically corrected for offset and gain by the following calculations:

$${\epsilon^{\prime }}_{i}=\epsilon_{i}-\epsilon_{0}-\left(\epsilon_{1023}-\epsilon_{0}\right)i/1023,$$ (1)

where

ϵ′*_i_* is the offset and gain corrected error at the *i*th code,
ϵ*_i_* is the measured error at the *i*th code,
*i* is the code index 0 to 1023,
*ϵ*_0_ is the measured error at the negative-most code, and
*ϵ*_1023_ low is the measured error at the positive-most code.

**Figure 4:**
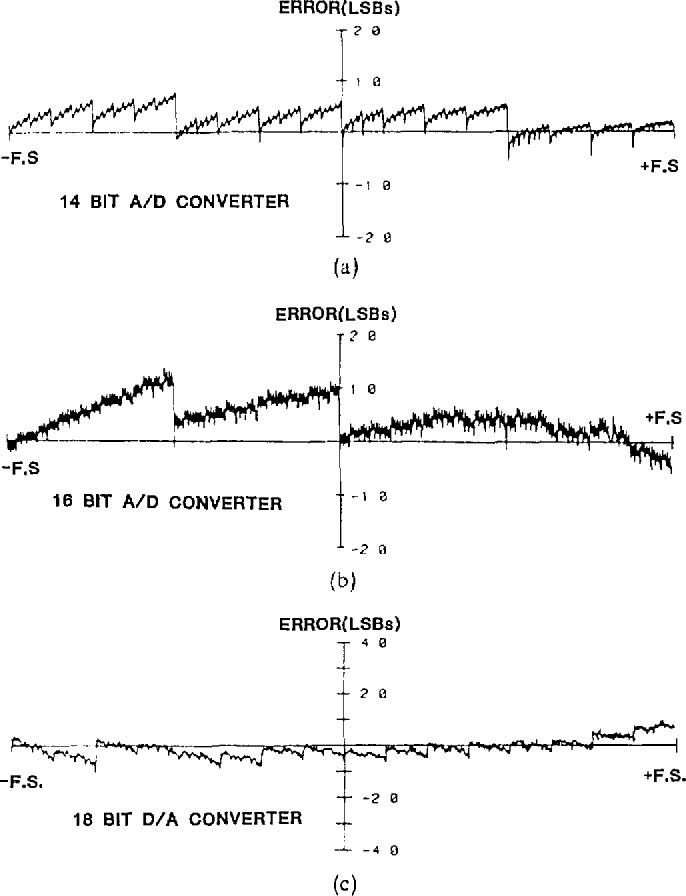
Typical linearity error plots. (a) 14-bit, 10-μs ADC. (b) 16-bit, 32-μs ADC. (c) 18-bit DAC, data average of five measurements.

The values of *ϵ*′*_i_* obtained from testing over the full scale range of the converter are used to determine the maximum, minimum, and rms value. Shown in figure 4 are some representative linearity error vs codeword plots obtained from the NIST static data converter test set. Keep in mind that these error plots show the *deviations* from an ideal transfer characteristic. Since differential linearity error (DLE) is defined as the error in separation between adjacent code transition levels, these errors are associated with the discontinuities observed in the plots of figure 4 (assuming as before that the bits less significant than the 10th contribute insignificant errors). Major discontinuities almost always occur at major code transitions; consequently, an abbreviated test is used to provide actual measurements of the DLEs at those 2(*n*-1) adjacent code pairs where the errors are likely to be the greatest. The DLE at each of these code pairs is simply computed as

$$\epsilon_{\mathrm{DL}}=\epsilon_{j}-\epsilon_{j-1},$$ (2)

where *ϵ_j_* and *ϵ_j_*_−1_ are the measured linearity errors at the selected adjacent code pairs.

### 2.2 Measuring Dynamic Transfer Characteristics

For most applications of data converters, the relationship between the analog and digital input/ouput variables under dynamic conditions is a primary property of concern. For DACs, this represents a deviation of the static transfer characteristic due to the inherent settling time of the DAC. Given a change in the digital input code, there will be a characteristic time required for the analog output of the DAC to settle to a new static level within some tolerance. Generally, the maximum settling time can be observed by giving the DAC a full scale code change at its input. This large change in the input will exercise the switching circuitry associated with the most significant bits of the converter, which are the critical ones in terms of settling time.

Making an accurate measurement of the settling time of a precision DAC to within 1 LSB, however, is not a trivial matter since it requires a good measurement of both amplitude and time. Methods for accurately measuring the settling time of DACs (as well as amplifiers and step generators) have been developed at NIST. These can be used to determine settling times as short as 10 ns to within a settling error of ±0.1% [15] (or as short as 1 μs to within a settling error of ±2 ppm [17]) for voltage output devices. Further improvements in the sampling voltage tracker system described in [15] are being pursued at the present time in order to improve the NIST capability to measure settling time accurately in the 1–10 ns region.

**Figure 5:**
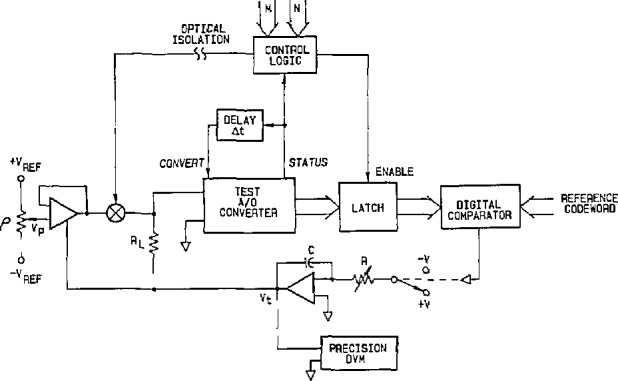
Simplified circuit diagram for the NIST dynamic ADC test set.

Dynamic errors in ADCs also are defined as any deviations from the static transfer characteristic that result from prior exercise, i.e., previous changes in input [5]. Since high-resolution (> 12-bit) ADCs in most applications are preceeded by an analog multiplexer or a S/H amplifier, exercising the ADC’s input with a fast-settling voltage step gives a good representation of the dynamic input conditions during actual use. An accurate method for providing stepped input voltage changes to ADCs for dynamic testing purposes was developed at NIST in the early 1980s [18]. Figure 5 shows a simplified diagram of the circuit used at NIST for dynamic ADC testing. It employs a programmable pulse level, *V*_p_, which is converted to a programmable current that is then switched (with optical isolation) through a low-value (200 Ω) resistor (R_L_) in series with the output of the feedback loop integrator. A programmable voltage step is thus superimposed on the transition level voltage established by the transition-locking feedback loop described above for static ADC testing.

**Figure 6:**
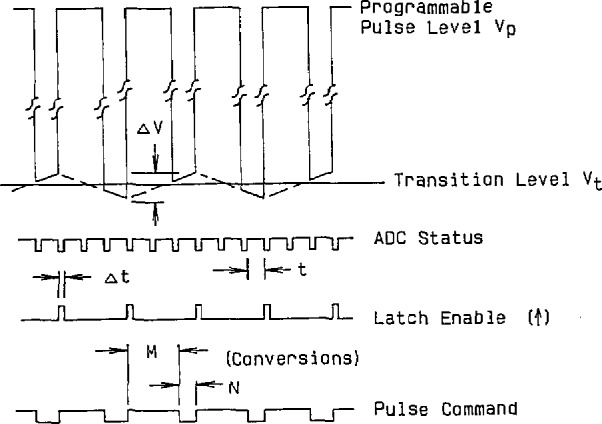
Timing diagrams, showing the relationships between the input voltage to the test converter (top) and three of the test set control signals.

Figure 6 gives some of the signal and timing diagrams associated with this test method, where the step changes to the programmed pulse level and back to the transition level are made between conversions, with time (*Δt*) allowed for the test ADC’s specified settling time. Although the test ADC converts when the input voltage is at either level, the ADC output samples (fed back to the digital comparator) are latched only for conversions made near the transition level. Any dynamic limitations of the test ADC (due to input buffer amplifier settling, internal DAC settling, delay in the internal analog comparator, etc.) will be manifest as a change in the static transition level that nominally would be maintained by the feedback loop. Therefore, the transition level maintained under the dynamic conditions just described is then measured by a precision digital voltmeter (DVM) and logged by the test set controller. Referring to the top waveform diagram in figure 6 (the input signal to the test converter), the portion of the signal about the transition level changes slowly, due to an adjustably long time constant of the integrator, with an amplitude given by

ΔV=(V/RC)(M+N)(t+Δt), (3)

where

*V* is the input voltage to the integrator,
*M* is the number of conversions made with the input at the programmable pulse level,
*N* is the number of conversions made with the input at the transition level,
*t* is the conversion time duration of the test ADC,
Δ*t* is the time delay to allow for the pulse to return to the transition level and the test unit to respond, and
*RC* is the time constant (*τ*) of the integrator.

Since the resolution of the transition level measurement is limited by Δ*V*, *R* (and, hence, *τ*) is adjusted to obtain an adequately small Δ*V* (nominally <0.1 LSB).

**Figure 7:**
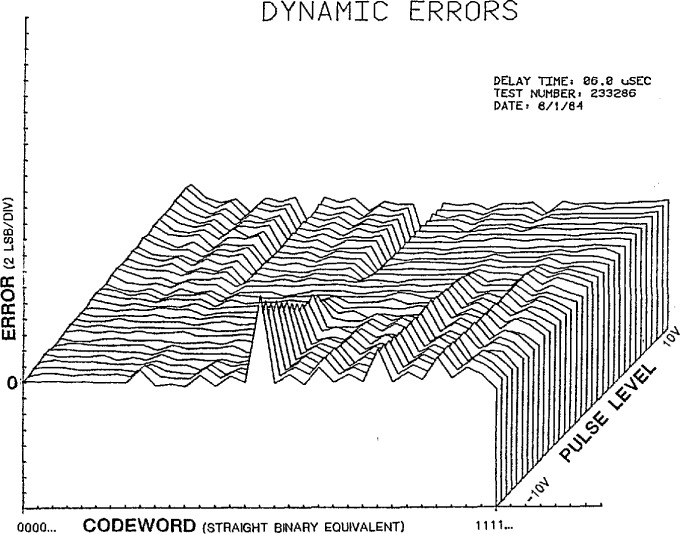
3-D plot of typical dynamic linearity errors for a 16-bit ADC.

Figure 7 shows a 3-D plot of the dynamic linearity error data obtained from a 16-bit ADC on the NIST dynamic ADC test set. The codeword axis is the measured code transition level and the pulse level axis is the range of possible pulse level amplitudes applied to the test ADC, with the vertical axis being the corresponding error in LSBs. This “surface” of dynamic bit errors is typical of the multi-bit errors that can often be observed, as compared to the fractional static LSBs typical of most ADCs (e.g., fig. 4). By varying the test set parameters (*M*, *N*, and Δ*t*) given in eq (3), one can investigate the cause of various features observed on the error surface [4].

## 3. Generating Reference Waveforms Digitally

**Figure 8:**
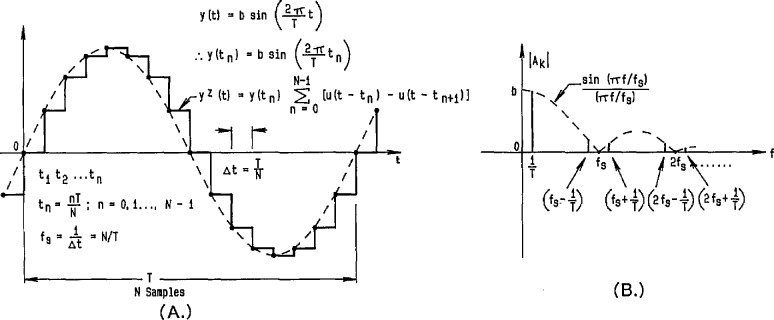
Time domain (a) and frequency domain (b) relationships of an ideal stepped (zero-order-hold) reconstruction of a sinusoidal waveform.

By making use of precision DACs that have small linearity errors (both statically and dynamically), reference waveforms can be generated by applying a time sequence of digital codes to the DAC and obtaining an analog output waveform consisting of steps corresponding to each of the input codes. Without further data processing on the input samples and the resultant output waveform, this stepped waveform is often referred to as a “zero order hold” reconstruction of the digital code sequence. Figure 8 (a) shows a representation of such an ideal stepped sinusoidal waveform in the time domain, with its corresponding line spectrum in the frequency domain given in figure 8 (b) (see the Appendix). Filtering out harmonics of the fundamental frequency located around multiples of the sampling frequency is relatively easy if the number of samples, *N*, is large enough so that the sampling harmonics are relatively small and fall outside the passband of the filter. The filtered output is then the original sine wave, only with its amplitude attenuated by the magnitude of the sin(*πf/f*_s_)/(*πf/f*_s_) function at the fundamental frequency. Of course, for imperfect filters and nonideal DACs, an actual line spectrum, such as the one shown in figure 8 (b), would have other small frequency components due to distortion and noise.

### 3.1 Audio Frequency Phase Angle Standard

**Figure 9:**
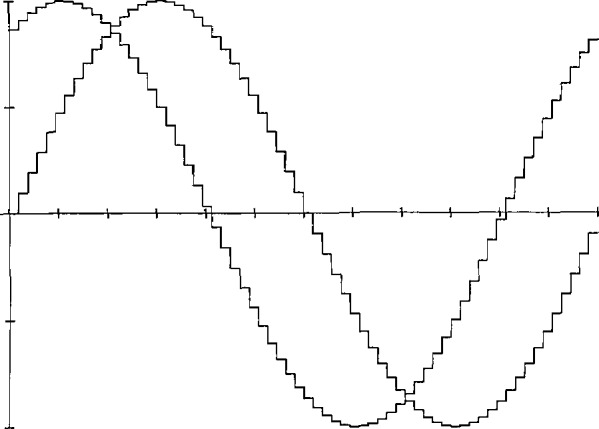
Stepped sine waves, phase difference 60°, 64 steps per cycle.

As illustrated in figure 9, by changing the time sequence of digital codes that are input simultaneously to a second DAC, a pair of stepped sine waves can be generated having a relative phase angle difference that is established essentially by the calculated sets of sample points, independent of the fundamental frequency and any other timing considerations used in the digital synthesis process. This is the basis for a precision 2 Hz to 50 kHz Phase Angle Calibration Standard that was developed at NIST [20,21]. An earlier version of this design approach was used for generating reference phase angle signals up to 5 kHz [6,7].

**Figure 10:**
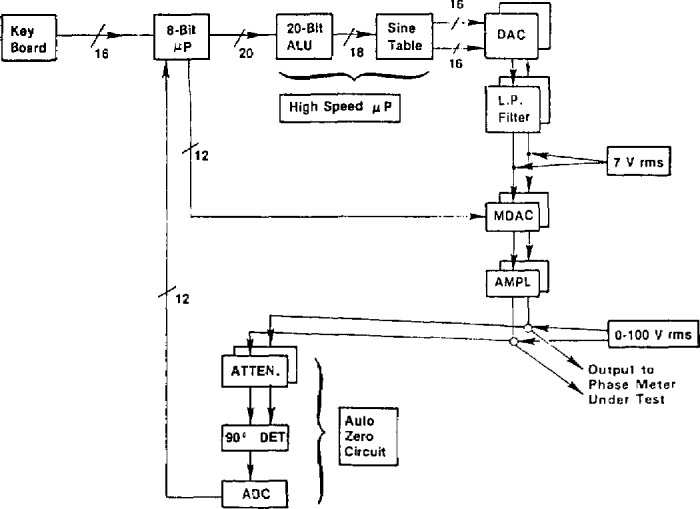
Block diagram of the NIST phase angle calibration standard.

A functional block diagram of the NIST Phase Angle Calibration Standard is shown in figure 10. A 20-bit, high-speed microprocessor (μP) computes the data needed for generating the 0–100 V rms output waveforms (delivered to a test phase meter). A slower 8-bit μP handles keyboard entries, correction data from the auto zero circuit, output scaling via the multiplying DACs (MDACs), and IEEE-488 interface and display functions (not shown). Eighteen-bit angle data (calculated by the 20-bit arithmetic logic unit (ALU) from phase and frequency keyboard entries) is fed sequentially to an angle-to-sine conversion module (sine table). Pairs of corresponding 16-bit amplitude data are then input simultaneously to the dual DACs through latches (not shown). The sample-point strobe pulses to the latches are generated by a crystal-controlled time base (also not shown). Thus, the waveforms that are output from the two DACs look like those of figure 9. These waveforms are then filtered to remove the unwanted steps (sampling harmonics), as mentioned above in conjunction with figure 8. Since most phase meters have voltage ranges that can accept input signals both above and below the 7 V rms output from the lowpass (L.P.) filters, the two sine waves are then scaled either up or down (from keyboard-entered amplitude data) by the MDACs and amplifiers. These scaling circuits cause small phase differences between the two output waveforms. To minimize these differences, a quadrature phase detection circuit is used to measure and compensate for the residual differential phase at 90°, thus establishing a fixed point on the phase angle scale.

The angular resolution of the phase standard depends on the number of sample points per cycle and on the amplitude resolution, corresponding to the number of bits of the DACs [20]. Each of the steps shown for the stepped sine waveform of figure 9 occur at a phase angle of

$$\phi_{n}=2\pi n/N,\;n=1,2,\ldots ,N$$ (4)

with an amplitude resolution of

$$\Delta V=2V_{p}/2^{M},$$ (5)

where

*N* is the number of samples per cycle,
*M* is the number of bits of the DAC, and
*V*_p_ is the peak value of the sine wave.

That is, at each step the phase angle can be set precisely, but the amplitude is limited to a quantized value within the resolution of the DAC. For *N*=64 and *M*= 12, ϕ_1_ = 5.625°, and Δ*V*= *V*_p_/2048. Because the amplitude steps are quantized, the phase angle between the output waveforms will also be quantized, although not necessarily uniformly. Thus, small changes in the programmed phase angle will not necessarily cause the phase difference between the two output waveforms to change. By incrementing *ϕ* a small amount at the *N* sample points until at least one sample pair (180° apart) changes by *ΔV*, the theoretically attainable phase resolution can be determined. Simulations have shown that for *N*=64, *M*=12, 97 percent of the possible phase increments will be less than or equal to 0.005° [20]. Below a fundamental frequency of 5 kHz where the phase standard uses 16-bit DACs, the theoretical resolution is better than 0.001°.

**Figure 11:**
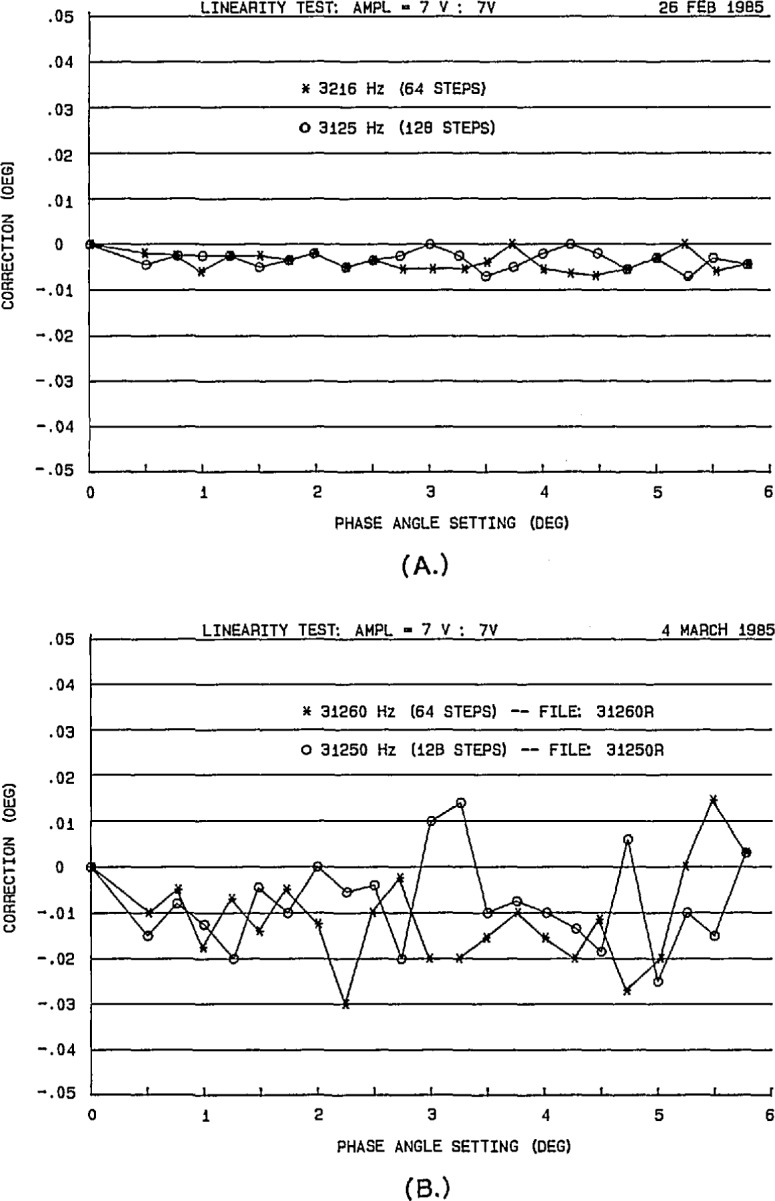
Measured phase linearity of the NIST phase angle calibration standard output (a) with 16-bit DACs at 3125 Hz, and (b) with 12-bit DACs at 31250 Hz (64 and 128 samples per cycle.

Because actual DACs are not perfectly linear and noise limits the uncertainty of the auto-zero compensation circuit, the NIST phase standard departs somewhat from its theoretical performance. Figure 11 shows the experimentally determined linearity for the phase standard using a 180° bridge [7,20]. The upper plot (a) shows the departure from the ideal 180° difference using 16-bit DACs at a frequency of 3125 Hz, generating the waveforms with both 64 and 128 samples per cycle. The lower plot (b) shows similar data obtained for the linearity using 12-bit DACs at a frequency of 31250 Hz. The range of the phase angle settings used for these plots is the basic 5.625° angle interval for *N*=64 and *M*=12. Using these and other experimental results, the maximum errors in the phase standard have been determined, as summarized in table 1.

With the development of the above-described NIST Phase Angle Calibration Standard, an automated calibration service for precision phase meters is now available [22].

### 3.2 Power Calibration Source

The traditional method of calibrating precision wattmeters and watt-hour meters is to compare the readings of the meter-under-test (MUT) to a standard wattmeter or watt-hour meter connected in parallel. However, it is advantageous, particularly in an automatic test setup, to be able to supply a separate source of voltage and current (“phantom” power) to the MUT where the source of voltage, and current (and the relative phase angle) is known and stable. Such a digitally synthesized power calibration source was developed at NIST to provide known parameters in order to test and calibrate a new class of multifunction instruments capable of measuring voltage, current, power factor, and active and reactive power [23].

**Figure 12:**
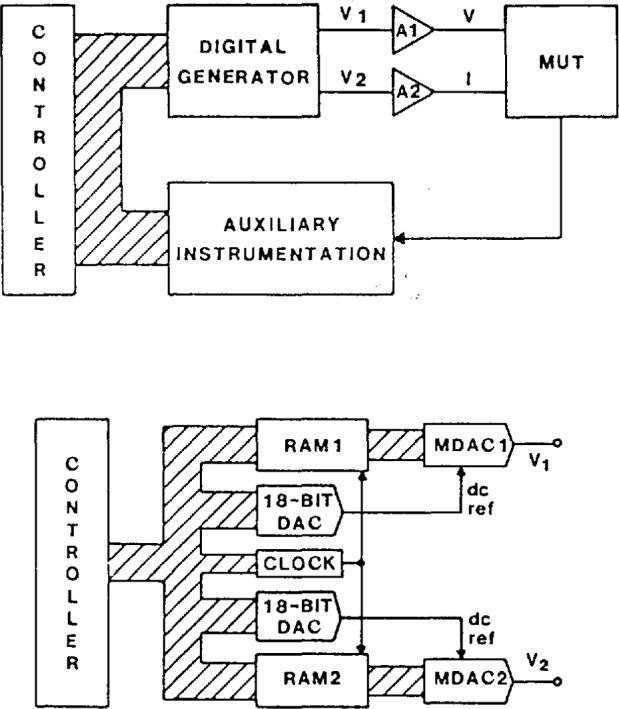
Block diagram of the NIST dual-channel power calibration source.

Figure 12 shows a block diagram of the NIST Power Calibration Source. The digital generator (shown in more detail in the lower part of fig. 12) is the heart of this source, and generates sine wave voltages V_1_ and V_2_ in a staircase approximation, as illustrated in figure 9. The waveforms in this case consist of 2048 steps per period at 60 Hz, which are then scaled by specially designed amplifiers A1 and A2 [24,25] to produce typical test voltages, *V*, ranging from 60 to 240 V rms, and typical test currents, *I*, ranging from 1 to 5 A rms. The relative phase angle between these voltages and currents applied to the MUT is determined essentially by the digital code sequences supplied to the MDACs from the random access memories (RAM1 and RAM2), similar to the technique used in the phase angle standard discussed above. In this generator, however, the codes to the MDACs are calculated by the controller (desktop computer), which uses sufficient numerical resolution to compute the sample points that roundoff or truncation errors in the phase angle are negligible. Consequently, the angular resolution using 16-bit MDACs is approximately 1 μrad (0.000057°) [23]. The amplitudes of V1 and V2 are independently set between 0 and 10 V peak by controlling the dc reference voltages supplied to MDAC1 and MDAC2 with a pair of 18-bit DACS. Consequently, the amplitude resolution for the power calibration source is 1 part in 2^18^ (about 4 ppm).

**Figure 13:**
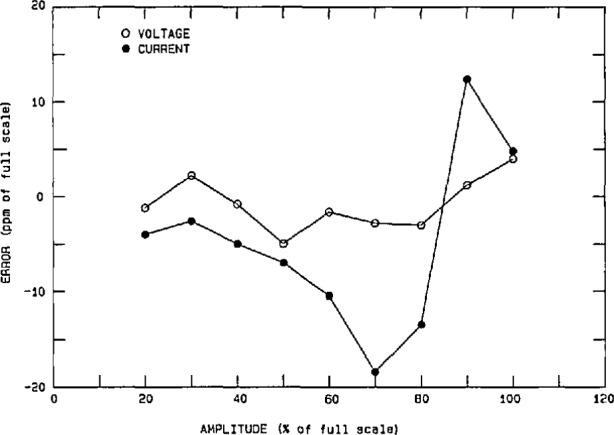
Integral amplitude nonlinearity in the power calibration source voltage and current outputs after gain corrections.

**Figure 14:**
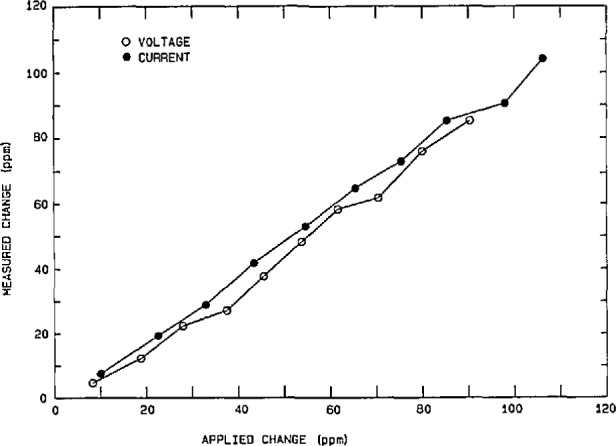
Differential nonlinearity of the voltage and current outputs around 120 V and 5 A rms.

**Figure 15:**
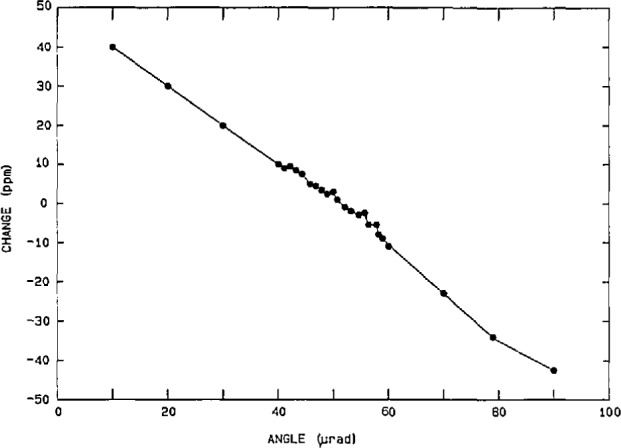
Phase differential nonlinearity: change in power indication of a TDM wattmeter (at zero power factor) versus change in phase angle from the NIST power calibration source.

**Figure 16:**
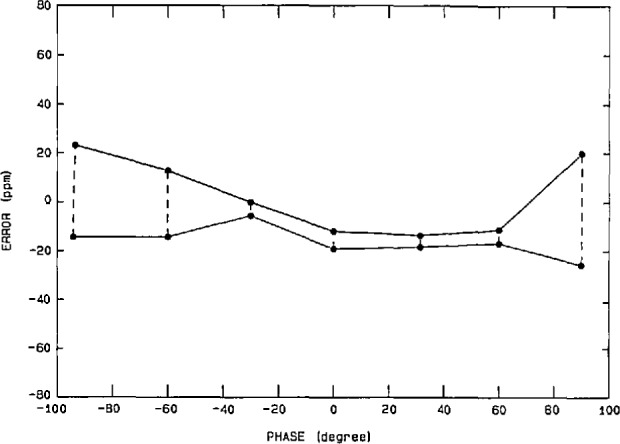
Maximum differences between the power calibration source and the NIST current-comparator power bridge, after correcting for current drift in the source.

The performance of the power calibration source (particularly at 60 Hz) has been measured using both a time-division-multiplier (TDM) wattmeter [26] as a transfer standard, and a current-comparator power bridge [27] as a ±15 ppm laboratory standard. Once again the nonlinearities, gain error, etc. in the characteristics of the actual DACs and amplifiers used in synthesizing the voltage and current outputs of the power calibration source limit its performance. However, software gain corrections for any amplitude (based on a linear fit to a few data points) help to reduce voltage-dependent gain error by a factor of 5 to 10. Figure 13 shows the integral amplitude nonlinearity (after gain corrections) in the voltage and current over a 5-to-1 range, using the current-comparator power bridge, where 100 percent of full scale represents 240 V rms and 5 A rms, respectively. Thus, we see residual uncertainties of <±20 ppm for current and <±10 ppm for voltage. However, differential linearity errors (about small changes in the output voltage and current at full scale) are in the 5 ppm level, as seen from figure 14. Figure 15 shows the phase differential nonlinearity at zero power factor (90°), as measured by a TDM wattmeter. This plot demonstrates the 1 μrad resolution of both the digital generator and the TDM wattmeter. Finally, by comparing the power calibration source with the NIST current-comparator power bridge, figure 16 shows the agreement in active power to be within a ±30 ppm band at power factors from zero lead to zero lag. The two curves represent an envelope of data points where the pair of dots connected by a dashed line at a given phase angle are the extremes of the measured errors.

### 3.3 Calculable RMS ac Voltage Source

For an ideal stepped sine wave, illustrated in figure 8(a), it can be shown that the rms value is identical to that of the corresponding pure sinusoidal waveform, i.e., *V*_p_/√2 [28]. However, the stepped waveform contains harmonics in its frequency spectrum [see fig. 8(b)], so that the unfiltered reconstruction contains harmonic distortion relative to the fundamental frequency component. Consequently, there is a tradeoff between the exact rms accuracy of ideal stepped waveforms (with their inherent harmonic distortion) and the rms error inherent in filtered reconstructions (that can have no distortion for a perfect low pass filter).

Total harmonic distortion (or, distortion factor) is defined as [29]

$$\begin{array}{l} \mathrm{THD}=\frac{\text{rms value of all harmonics}}{\text{rms value of all fundamental}},\\ \;={\left(\sum_{i=2}^{\infty }{A^{2}}_{i}/{A^{2}}_{1}\right)}^{1/2}, \end{array}$$ (6)

where *A_i_* is the peak value of the *i*th harmonic, computed from

$$A_{i}=V_{p}\left[\sin\left(\pi f_{i}/f_{s}\right)/\left(\pi f_{i}/f_{s}\right)\right],$$ (7)

*V*_p_ is the peak value of the sine wave,
*f_i_* is the *i*th harmonic frequency, and
*f*_s_ is the sampling frequency.

For comparison of the tradeoff between the rms error and THD between two sine wave reconstructions, table 2 shows these relationships for a stepped sine wave composed of 20 steps and another composed of 200 steps. It also shows a comparison with perfectly filtered (i.e., “brick wall”) reconstruction.

An ac reference standard was developed at NIST in the early 1980s that used zero order hold digital waveform synthesis to produce both filtered and unfiltered sine wave reconstruction [30]. A built-in, rms-to-digital converter that used a thermal voltage converter (TVC) and voltage-to-frequency ADC was used to measure the rms value of the output waveform and provide error corrections.

Uncertainties of ±50 ppm were achieved in the 0 to 7.07 V rms output voltage over a frequency range of 1 Hz to 50 kHz (with only 0.5% THD at 50 kHz using a lowpass, active filter). This ac reference is very useful for calibrating narrowband, rms-responding voltmeters having a ±0.01% accuracy specification.

However, new wideband voltmeters (and most thermal voltage converters) can respond to the harmonics in stepped waveforms, and still claim rms measurement accuracy below 0.01% (100 ppm). To provide a transportable ac source for these voltmeters at an accuracy of ± 10 ppm over a 30 Hz to 15 kHz range, a digitally synthesized source (DSS) using zero order hold reconstruction has been developed [31]. As indicated above (and shown in table 2), ideal stepped sine waves have no rms error and are, therefore, desirable rms reference waveforms.

**Figure 17:**
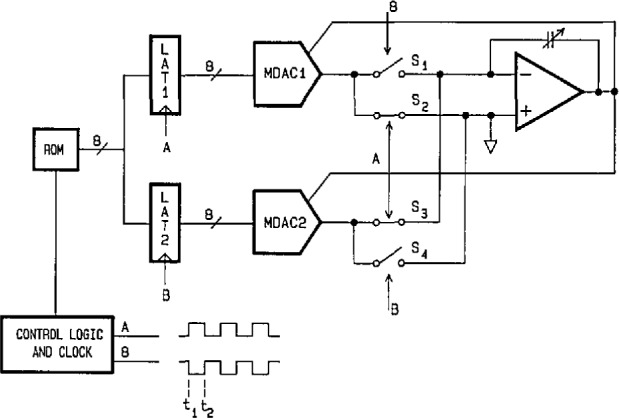
Simplified diagram of the circuit used in the dual-DAC, digitally synthesized source (DSS).

Figure 17 shows a simplified circuit diagram of the dual-DAC synthesis approach used in the DSS. Its general design is similar to that of the generating circuits used for the phase angle standard and the power calibration source (see figs. 10 and 12). In addition, a “deglitching” technique (used to improve phase angle precision [32]) is employed in the DSS to enhance the quality of the generated steps. By toggling between the output currents of the two MDACs, which are updated with data from the latches at different phases of clock signals A and B, the output voltage steps from the amplifier are not subject to code-dependent transitions and settling-time errors from the MDACs. From t_1_ to t_2_, MDAC2 supplies the output step (via switch S_3_ and the output amplifier) while MDAC1 is settling to the level corresponding to the input code. At time t_2_ new data from the ROM is latched into MDAC2, S_1_ and S_4_ are closed (S_2_ and S_3_ are opened), and MDAC1 now supplies current for the next output voltage step. Obviously, successive data are clocked from the ROM into the latches, alternately, at twice the rate of clocks A and B.

**Figure 18:**
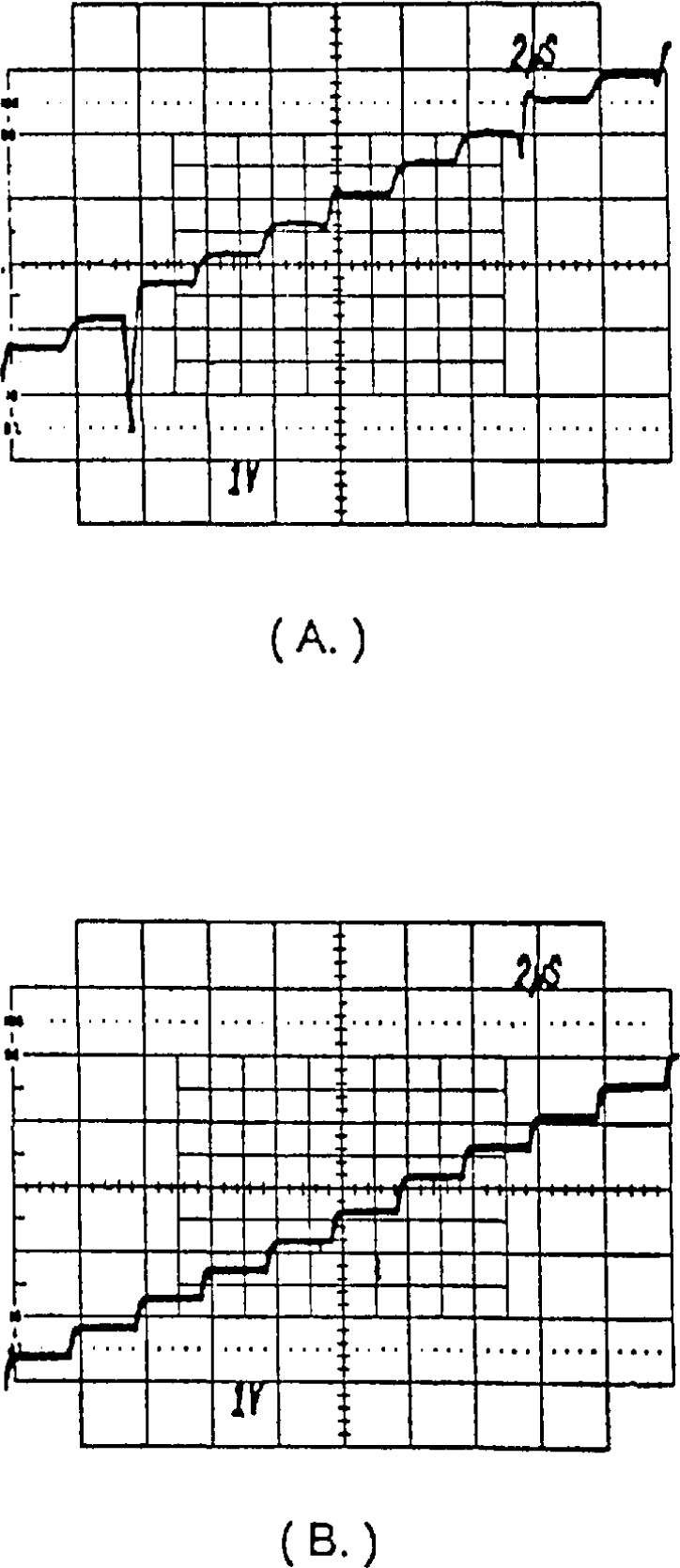
Oscilloscope traces of an unfiltered portion of a 28-step digitally synthesized sine wave (1-MHz clock rate), (a) Using a single DAC. (b) Using the dual-DAC deglitching technique.

Of course, imperfect steps still remain due to small amounts of strobe feedthrough from the (fast CMOS) switches and finite bandwidth and slew rate limitations in the amplifier. However, as seen in figure 18, the improvement in the fidelity of the steps using this technique is significant. The major code transition glitch seen in figure 18 (a) and other structural differences between the steps are virtually eliminated in figure 18 (b).

The rms voltage of the waveforms generated by the DSS over its nominal frequency range is calculated by stepping through the sample points stored in the ROM at a low speed in order to measure the amplitude of each step with a high-accuracy DVM. The estimated rms value of the waveform using this “step calibration” is determined by computing the square root of the mean value of the squares of the measured step voltages, i.e.,

$$V_{\mathrm{rms}}=\left[\sum_{i=1}^{N}[v_{i}{\left(1+C\right)]}^{2}/N\right]{\;}^{1/2},$$ (8)

where

*v_i_* is the voltage of the *i*th step
*N* is the number of steps in one period, and
*C* is the DVM correction.

In order to assure that the estimate is good to a few ppm, the DVM nonlinearity must be only a few ppm of full scale and have similar short term stability during the test time. This computed rms value then will be valid for low- frequency waveforms, but will degrade at higher frequencies as imperfections due to transients become a larger portion of each of the steps.

**Figure 19:**
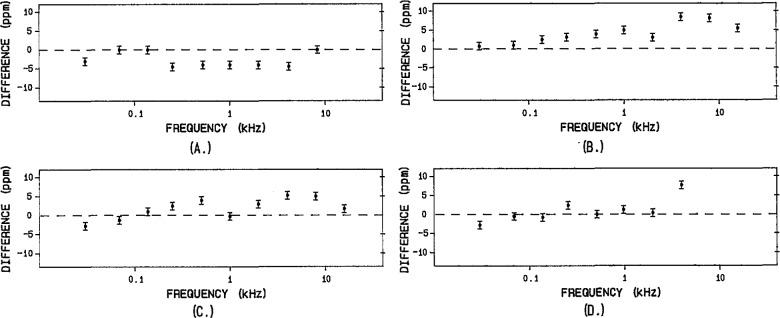
The difference in ppm between the rms value computed by the step calibration (dashed line) and the rms value measured using thermal techniques: (a) DSS1 using the internal clock to generate a sine wave, (b) DSS2 using the internal clock to generate a sine wave, (c) DSS2 using an external clock to generate a sine wave, (d) DSS2 using an external clock to generate a (nonsinusoidal) waveform.

The rms values of the outputs from two of the DSS prototypes have been measured at NIST with coaxial TVC standards at sine wave frequencies between 30 Hz and 15 kHz. The results of these step and thermal measurements are given in figure 19 for two different units (DSS1 and DSS2) using both internal and external clocks to synthesize 128-step sine wave approximations (A, B, and C). As a further demonstration, the results of measurements on a nonsinusoidal waveform (sine wave approximation with 30% third harmonic) are shown in D. The plots show the average difference between three sets of measurements consisting of two step calibrations (represented by the dashed line) and five thermal measurements (plotted points with one standard deviation error bars) at each frequency. Agreement between the two measurements is better than ±5 ppm from 30 Hz to about 2 kHz. A small capacitor on the output amplifier (see fig. 17) is initially adjusted to obtain flatness of the rms value to within ±10 ppm from 2 to 15 kHz. Recent designs have now extended this performance up to 50 kHz.

## 4. Measuring Waveform Parameters Using Digital Sampling

**Figure 20:**
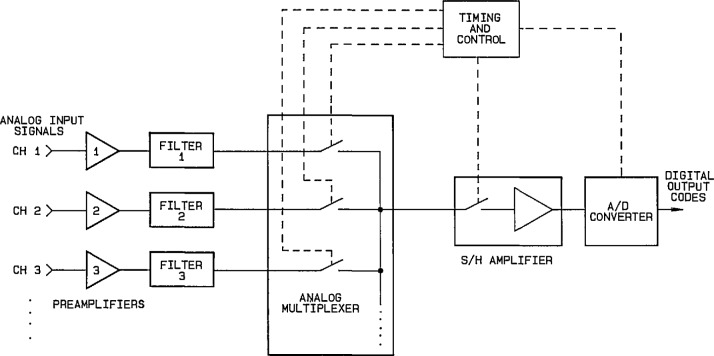
Block diagram of a typical multichannel sampling measurement system.

To make accurate measurements of signal waveforms that can be stored and processed further with a computer requires the use of precision ADCs having small linearity errors (both statically and dynamically), as described above. In order to convert fast signals that change during the conversion time of the ADC, a sample-and-hold (S/H) or track-and-hold (T/H) circuit is inserted between the signal source and the ADC to capture samples of the waveform and hold the ADC input signal at a steady level while the ADC completes a conversion cycle. Often there are also signal conditioning amplifiers and filters, as well as multiplexing switches, employed in a complete data acquisition channel. Figure 20 shows a block diagram of a typical multichannel measurement system. Analogous to the reconstruction of analog waveforms using a time sequence of digital input codes to a DAC (described earlier), the process of digital waveform sampling generates a time sequence of digital output codes from an ADC, corresponding to the amplitude levels of the analog input waveform at the sampling instants.

In order to understand the limitations imposed by using digital sampling methods to measure the parameters of signal waveforms, a short discussion on the basics of sampling theory is provided in the Appendix.

### 4.2 Low-Frequency ac Sampling Voltmeter

The speed and versatility that is possible with digital waveform sampling of low-frequency ac waveforms was demonstrated with the development of the NIST Low-Frequency ac Sampling Voltmeter [35]. An rms low-frequency (0.1 to 50 Hz) voltmeter/calibrator had been developed at NIST about the same time, and employed a multi-junction thermal converter device for making accurate rms-to-dc conversions. This instrument is capable of making ac transfer measurements (comparing ac input voltages against its internal calibrator), good to ±0.02% accuracy [36]. The thermal converter-based instrument serves as the standard in a NIST calibration service for ac voltmeters and calibrators from 0.1 to 10 Hz [37]. With the sampling-based voltmeter, rms measurements good to ±0.1% accuracy at 10 mV to 10 V levels from 0.1 Hz to 120 Hz are available, as are measurements of the input signal frequency, total harmonic distortion (THD), and the rms value of the ac component (only). Also, the sampling instrument uses “window crossing” and error function algorithms in the software of its internal microcomputer to permit measurements within two signal periods at frequencies below 10 Hz [38]. Thus, measurements of a 0.1 Hz signal can be made in 20 s (2 s for a 1 Hz signal), which is 2–10 times faster than the thermal converter-based instrument.

**Figure 21:**
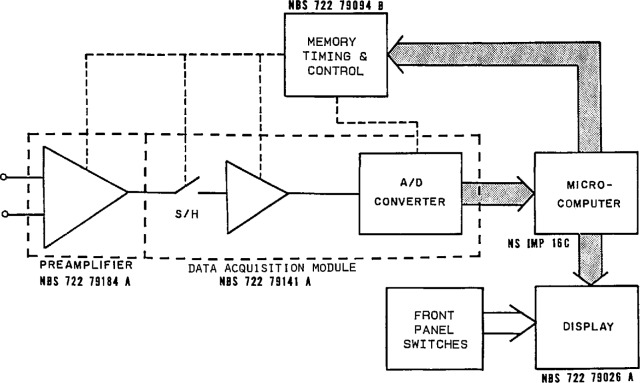
Simplified block diagram of the NIST low-frequency sampling voltmeter.

Figure 21 shows a simplified block diagram of the low-frequency sampling voltmeter. Functionally similar to the measurement system shown in figure 20, this instrument employs a low-noise, differential-input preamplifier with programmable gain-setting resistors, so that four decades of gain can be programmed from the microcomputer via the memory, timing, and control section. A data acquisition module consisting of an S/H amplifier, an instrumentation amplifier, and a 12-bit ADC (11 bits plus sign bit) provides the sampling, signal conditioning, and digitizing of the preamplifier output signals. With the three programmable input voltage ranges of the data acquisition module, a total of 12 gain settings are available using combinations of preamplifier gain and data acquisition range settings. The memory, timing, and control block contains the instruction program (2048 16-bit words) stored in read-only-memories (ROM), as well as a small amount of random-access-memory (RAM) for temporary storage of input values and program variables. The 16-bit microcomputer used for this application was chosen for its powerful microprogrammable instruction set that allowed straightforward implementation of the multiplications used in computing the fast Fourier transform needed for calculating THD.

**Figure 22:**
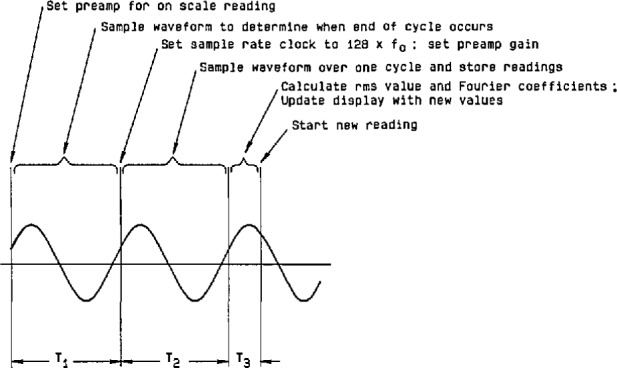
The time sequence of the measurement cycle in the voltmeter, relative to a typical sinusoidal input.

**Figure 23:**
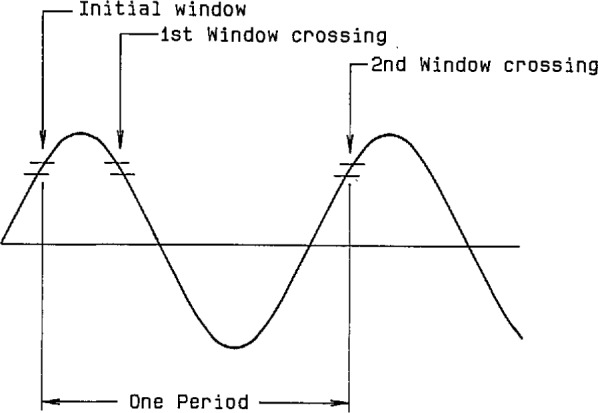
Approximate period detection using window crossing.

Because the algorithm for calculating rms value requires an accurate value for the period of the sinusoidal input signal, the initial sampling sequence is used to obtain an approximate value for the period (see fig. 22). During the first cycle (T_1_), sampling is begun at any arbitrary point on the waveform, after possible adjustment of the preamplifier gain. Since the frequency may be as low as 0.1 Hz or as high as 120 Hz, a decision is made, based on the slope of the signal, whether a fast (8 kHz) or slow (500 Hz) sampling rate will be used to find the approximate period. With the sampling rate set at 500 Hz, an initial reading is taken; a higher and lower threshold value is then calculated with respect to this initial reading. Thus, an “initial window” of sample values is established, as shown in figure 23. Depending on the number of samples it takes to cross the upper or lower threshold, the sampling rate continues to be 500 Hz, or else the sampling rate clock is reset to 8 kHz, and the measurement sequence is restarted. After the waveform is sampled further, and a “2nd window crossing” is found, approximately one signal period has elapsed. The sampling rate clock is then set for 128 times the approximate frequency (f_0_), the reciprocal of the approximate period, and sampling continues for slightly more than one period (cycle T_2_).

To determine the signal period more exactly, an autocorrelation technique is performed using sample points taken during T_2_. To take possible effects of noise and frequency modulation (FM) into account, 147 samples are taken during T_2_. The correlation is then accomplished by evaluating an error function, *E*_f_, that consists of summing the difference in the values of samples taken at approximately corresponding times in the cycle:

$$E_{f}=\sum_{i=1}^{n}\left|E_{i}-E_{i+N}\right|$$ (9)

where

*N* is the variable number of samples in period (125, 126, 127, 128, 129, 130, and 131),
*E_i_* are sample values at the beginning of the cycle,
*E_i+N_* are corresponding values at the beginning of the next cycle, and
*n* is the number of difference values summed; 16 points chosen, based on empirical testing.

If the period was exactly an integral number of samples and the input signal was free of noise or FM, this sum would go to zero for *N* = 128. Since this is rarely the case, *N* is varied from 125 to 131 until a minimum value for the error function is found. However, because this error function can exhibit an undesired second minimum that can be difficult to distinquish from the true minimum, the two smallest sums of the seven error functions of (9) are used to linearly interpolate to the minimum point between them [38].

The rms value of the input is then calculated in a straightforward manner from the appropriate set of stored samples. The sample values are squared and summed using 32-bit precision arithmetic. For the “ac only” feature of this instrument, the mean squared value is calculated as usual, and then the square of the average of the input samples (the dc component) is subtracted before taking the square root for the net ac rms value.

To obtain the dc, fundamental, and first 30 harmonic components of the input signal, the data samples from the second cycle (T_2_) are used to calculate the coefficients of the discrete Fourier transform (DFT) of the signal. This calculation is accomplished by using a 64-point radix 2 fast Fourier transform (FFT) algorithm. The magnitudes of these coefficients are then used to calculate the THD as a figure of merit of the signal as a pure sine wave. However, this instrument is designed mainly for measuring low-distortion sinusoidal signals, with the highest computed harmonic being

$$f_{\max}=30f_{0},$$ (10)

so that

$$f_{s}=128f_{0}\geq 2f_{\max},$$ (11)

thus more than satisfying eq (15A) (see the Appendix) for the Nyquist frequency criterion to prevent aliasing.

### 4.3 Wideband Sampling Wattmeter

With the widespread use of solid-state thyristor switching devices for controlling the delivery of electrical power, the need has grown for making accurate measurements of highly distorted power signals. To meet this need a wideband sampling wattmeter was developed. This wattmeter uses asynchronous sampling of its input waveforms to realize a measurement uncertainty of less than ±0.1% of the full scale range for fundamental frequencies from 1 Hz to 10 kHz (and harmonics up to 100 kHz) [14]. A prototype instrument, constructed in the mid 1980s, is programmable from the front panel and has-the necessary hardware and software to optimize the sampling frequency and to make corrections for truncation errors [39]. In the early 1970s the feasibility was demonstrated for making precision ac power measurements (±0.02% accuracy up to 2 kHz) using a sampling technique, and computing the power with a suitable algorithm [40].

In contrast with the synchronous (phase-locked) sampling method used in the earlier sampling wattmeter (and the correlation approach used in the low-frequency ac sampling voltmeter described above), asynchronous sampling of the two input (voltage and current) waveforms is used in the wideband sampling wattmeter. That is, the average power for the periodic input signals is calculated by using the approximation given by

$$W=1/n\sum_{k=0}^{n-1}V(t_{k})I(t_{k}),$$ (12)

where

*V*(*t_k_*) and *I*(*t_k_*) are the uniformly spaced samples,
*n* is the total number of samples in the record, and
*W* is the indicated average power.

**Figure 24:**
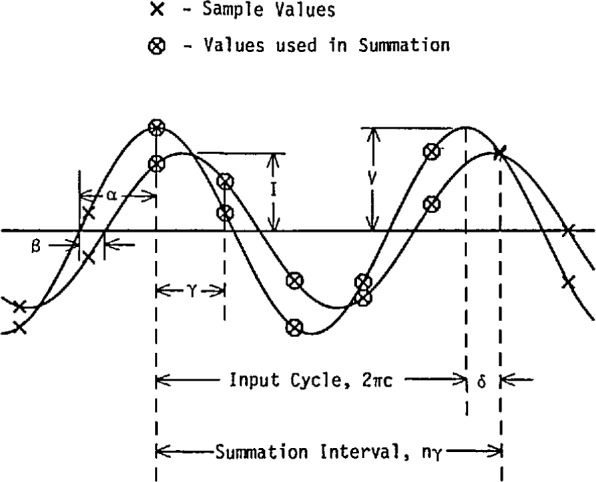
Asynchronous sampling of voltage and current sine waves showing the relationship of various sampling parameters. In this example, *c* = 1, *n*=5, *α*=90°, *β*= − 30°, *τ*=80°, and δ=−40°.

The sampling times, *t_k_*, are not synchronized with the period (i.e., frequency) of the input signals. Consequently, the interval over which the summation is taken does not coincide with an integral number of cycles of the input signals. Figure 24 shows the relationship between the sample times and the associated voltage and current signals. To calculate the power in such signals, the sample values from a finite interval (the summation interval) of the waveforms are used, as indicated by the circled sample values. With the sample interval, γ, expressed in radians, the voltage and current samples are given by

$$V\left(t_{k}\right)=V_{k}=V\sin\left(k\gamma +\alpha \right)$$ (13)

and

$$I\left(t_{k}\right)=I_{k}=I\sin\left(k\gamma +\alpha +\beta \right),\;k=0\;\mathrm{to}\;n-1$$ (14)

where

*κ* is the sample number,
*α* is the starting voltage phase angle,
*β* is the relative phase angle between the voltage and current, and
*V* and *I* are the peak values.

For a signal of period *T* seconds and a sampling rate of *r* samples per second,

γ=2π/rT. (15)

Using eq (13), the approximate average power is then,

$$W=\frac{VI}{2}\left[\cos\beta -1/n\sum_{k=0}^{n-1}\cos\left(2ky+2\alpha +\beta \right)\right].$$ (16)

The true power, *P*, is just the first term in this expression, so that the second term is the error due to the nonintegral number of samples in the period (i.e., truncation error, *E*). The difference between the summation interval, given by *nγ*, and the nearest integral number of cycles, *c*, of the input signal is defined as the truncation angle, δ; that is,

δ≡2πc−nγ. (17)

The truncation error then can be shown to be expressed as [14]

$$E=\frac{VI}{2n}\frac{\sin\delta }{\sin\tau }\cos\left(2\alpha +\beta -\delta -\gamma \right).$$ (18)

**Figure 25:**
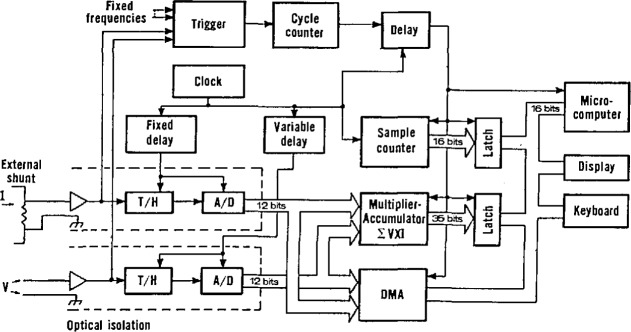
Block diagram of the NIST wideband sampling wattmeter.

This expression shows that if the truncation angle, δ, is zero, then *E* is zero, which is the reason why many sampling wattmeters use synchronous sampling. Although the wideband sampling wattmeter described here has asynchronous sampling, it uses trigger and cycle counting hardware, as shown in the block diagram of figure 25, to control the summation interval. By starting and stopping the summation interval with a trigger pulse synchronized with the signal being measured, the truncation angle, δ, is kept to less than the sample interval, γ. For γ≤90°, eq (18) shows that the maximum error occurs when sin δ=sin γ and the cosine term is ±1. Then, the maximum error is given by

$$E_{\max}=\pm \frac{VI}{2n},$$ (19)

showing that the maximum truncation error can be made small by taking a large number of samples, *n*, independent of the input signal frequency. With the NIST wideband sampling wattmeter, power measurements are typically made with an *n* of greater than 150,000 samples so that the truncation error is negligible. With a sampling rate of 300 kHz, this number of samples requires a summation interval of about one-half second.

With the truncation error due to asynchronous sampling minimized, the remaining measurement errors are a combination of the errors in the scaling amplifiers, the track and hold (T/H) amplifiers, and the 12-bit A/D converters shown in figure 25. The T/H and ADC modules were tested with the NIST static data converter test set described previously and found to have rms linearity errors <±0.5 LSB, or <±0.012% of full scale. Differential time delays between the two input amplifiers (as much as 46 ns) are compensated to within 1 ns by the programmable fixed and variable delay units that control the time when the convert command signals are applied to the T/H and ADC modules. The need to correct for this error can be illustrated by considering that if the wattmeter is measuring a 10 kHz power signal at half-power-factor, i.e., a phase angle of 60°, then a differential time delay of only 18 ns could cause an error of ±0.2% of the true power value.

**Figure 26:**
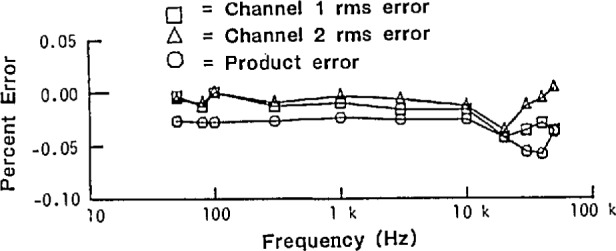
RMS error (in percent) versus frequency for each channel and for the product (simulating a unity power factor input signal).

Figure 26 shows the percent rms error of the NIST wideband sampling wattmeter as a function of frequency (on the 1 V range), determined by comparing its measurements results with those from an ac voltmeter whose frequency response up to 100 kHz had been established using NIST-calibrated thermal voltage converter standards. The error in both the rms value of each channel and the product (simulated power) function is well within the desired goal (±0.1%), even out through 50 kHz. The results for the 0.1-, 0.2-, and 0.5-V ranges are better than these, and those for the 10- and 500-V ranges using a specially-designed 100-to-1 attenuator are poorer by a factor of about two. Comparison tests made with a standard thermal wattmeter [41] showed agreement to within ±0.03% of full scale over the frequency range from 40 Hz to 2 kHz [14]. Since distorted waveforms can be decomposed by Fourier analysis into harmonically-related sine waves, and assuming that linearity (and, hence, superposition) holds, the performance of the wideband sampling wattmeter for measuring distorted waveforms can be predicted by using the errors shown in figure 26.

### 4.4 Sampling Voltage Tracker

A third method of accurately sampling and digitizing repetitive waveforms has been employed at NIST in the development of a sampling voltage tracker (SVT) system. This system is capable of making state-of-the-art equivalent-time measurements for signals with frequency components up to 200 MHz [15]. The rf rms voltage measurement accuracy of this system has been shown to be comparable to that of thermal voltage converter standards [42].

The equivalent-time sampling process implemented by the SVT is somewhat different from that illustrated in figure 38 (see the Appendix). The SVT is constrained by its design to sample each point on the waveform numerous times (over as many waveform periods), and the data processed to estimate the value at that point on the waveform. Then, the time base is incremented one sampling interval to acquire and process samples for the next point, and so on, until the entire sequence of estimated sample values is obtained. It should be noted that no large amount of memory is required by this method in order to perform the averaging process.

As with any sampling system, timing jitter affects the measurement accuracy of the SVT. However, unlike many sampling systems, the SVT is insensitive to timing jitter over all locally monotonic regions of the input waveform. This property results from the statistical estimator that is used by the SVT to deduce the “true” sample values from many time-jittered observations—the so-called Markov estimator. It has been investigated [43] and found to be equivalent to the median estimator, which is known to be unbiased for monotonic functions. On the other hand, the sample mean, which is typically used in most sampling systems, is generally biased for any function that has second or higher order derivatives.

**Figure 27:**
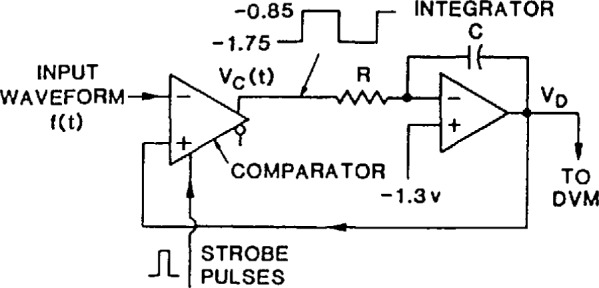
Basic circuit of the NIST sampling voltage tracker.

**Figure 28:**
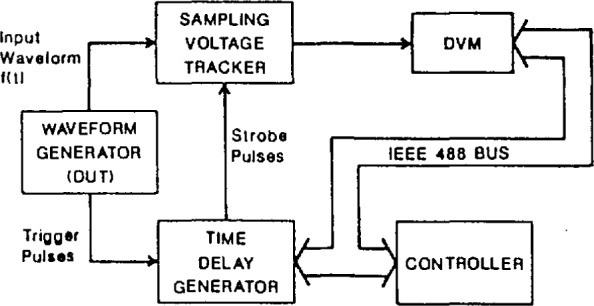
Block diagrams of a complete SVT system.

Figure 27 illustrates the basic components of an integrating feedback amplifier SVT circuit, of the type used in the NIST SVT system. The analog comparator is triggered by strobe pulses to make successive comparisons of the input waveform amplitude with that of the feedback signal from the integrator, at a given instant on the input waveform. The feedback signal, obtained by integrating the emitter-coupled logic (ECL) level waveform from the output of the comparator, is an estimate of the amplitude at a given point on the input waveform. When the average (dc) level of the feedback signal amplitude matches that of the input, the comparator output is a square wave. Consequently, the output of the integrator is a triangle wave whose peak-to-peak amplitude is made relatively small by adjusting the RC time constant of the integrator. As shown in figure 28, a precision DVM is used to measure the average value of the integrator output waveform. The process is repeated at successive points, determined by a precision time-delay generator, until the entire input waveform has been sampled and the computer/controller has reconstructed the waveform in equivalent-time.

In order to use the NIST SVT as an accurate waveform measurement tool, its performance as a precision sampling system has been characterized in terms of static and dynamic linearity errors, time base errors, and step and frequency response errors [15].

#### 4.4.1 Linearity Errors

**Figure 29:**
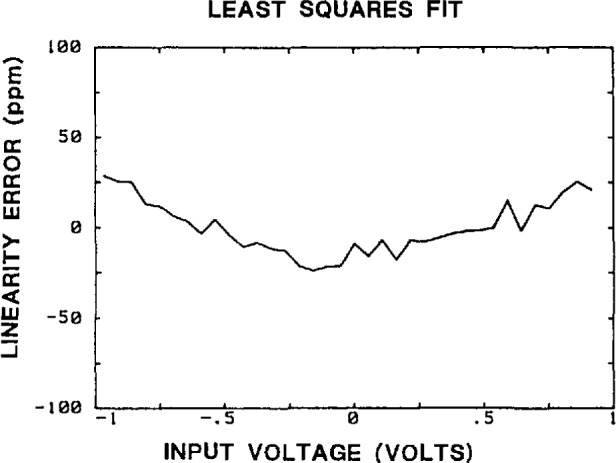
Typical plot of the SVT linearity errors.

Figure 29 shows a typical plot of the static linearity error obtained by supplying known, programmable dc voltages to the SVT input terminals, recording the readings, and performing a linear regression of the form

**Figure 30:**
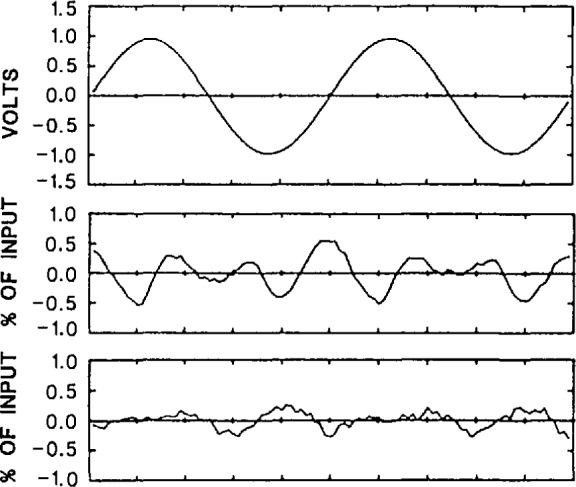
Typical plot of a digitized 50 MHz sine wave (top) and dynamic linearity errors derived from curve fitting for 1-V peak input (middle), and 50-mV peak input (bottom). The horizontal scale is 4 ns per division.

Y=ax+b (20)

on the results. The residuals of the fit are the static linearity errors. Dynamic linearity errors (or harmonic distortion) are measured by inputing high purity sine waves, and fitting an ideal sine wave to the recorded samples. The residuals of the fit in this case include not only the amplitude linearity errors, but also errors due to nonlinearities in the time base (which are minimized by using an averaging technique explained in the next paragraph). Figure 30 shows typical plots of the dynamic linearity errors for 1 V and 50 mV sine wave input signal amplitudes, at a frequency of 50 MHz; similar data were obtained for other frequencies. The rms linearity error versus frequency, expressed as a percentage of the rms value of the fitted sine wave, can be found in table 3 (together with other characteristics that summarize the performance of the NIST SVT system). Note that the linearity errors (given as a percentage of the applied signal) generally decrease at lower signal levels.

#### 4.4.2 Time-Base Errors

**Figure 31:**
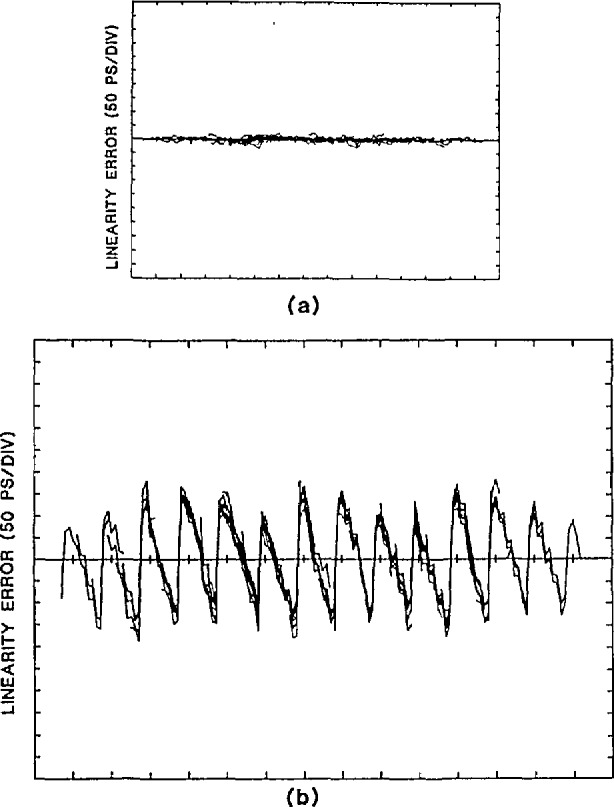
Time-base linearity errors for (a) and (b): two different time bases. The errors shown in (a) result from ten different data sets overlaid for each plot. In (a) the linearity errors are buried in noise. The horizontal scale is 10 ns per division.

Nonlinearities in the time base cause displacements of the sampling instants, which in turn cause apparent amplitude errors proportional to the derivative of the signal being measured. By incrementally shifting the phase of the input sine-wave signal relative to the trigger pulse, a record is obtained in which the time-base errors are shifted with respect to the phase of the sine wave. This process is repeated *m* times. The records are then realigned in phase and averaged point-by-point, which requires two periods of the signal. The time-base errors can be estimated by subtracting the averaged data, in which the time-base errors have been filtered out, from the nonaveraged records, and dividing by the derivative of the signal at each sample point (regions near the cusps of the sine wave where the derivative approaches zero, of course, must be avoided). The results of such measurements are given in figure 31 for two different time bases. In both cases the period of each record was 100 ns (two cycles of the 20-MHz test sine wave), the equivalent-time sampling interval was 1 ns, and the *m* = 9 shifted records provide up to 10 estimates per sample point. The time base used for figure 31(a) has small random errors of only 7 ps over the ~ 140 ns time epoch; figure 31(b) is an example of a time base where the linearity errors are much more evident. The periodicity of these errors is due to the interaction of the separate coarse and fine adjustment circuitry used in this time base.

Another significant source of time-base errors is the result of timing jitter (phase noise) between the strobe pulses and the input signal. Phase-noise components in the measured signal, the trigger signal, and the time-delay generator all contribute to the overall phase-noise error. The low-frequency components of this noise cause relatively slow variations in the integrator feedback signal; these can be observed by monitoring the feedback signal on an oscilloscope. With the delay set to sample the input waveform at a point where the rate of change is high, amplitude fluctuations in the feedback signal resulting from phase noise are easily observed with the scope set for high sensitivity and ac coupling. The observed peak amplitude is the product of the peak phase displacement (expressed in units of time) and the time rate of change of the input signal at the nominal sampling instant. For low phase-noise signals, the equivalent timing uncertainty measured in this way on the NIST SVT was less than 10 ps (peak).

#### 4.4.3 Step and Frequency Response Errors

**Figure 32:**
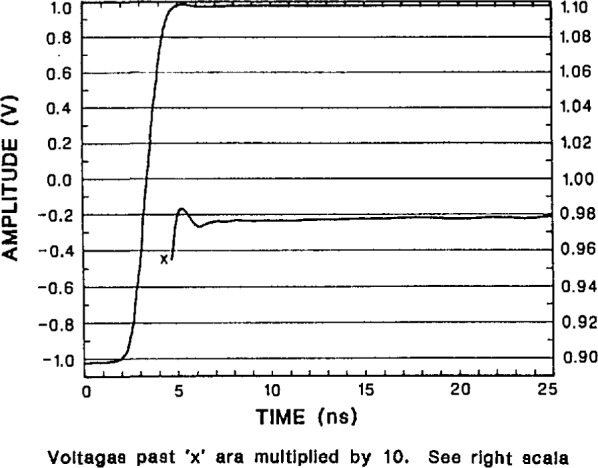
Response of the NIST SVT to a 2-V step from the NIST programmable step generator, showing settling to within ±0.1% of full scale range (FSR) in 4 ns. Note that the lower curve is a magnified version of the final settling, with the scale given on the right hand side of the plot.

**Figure 33:**
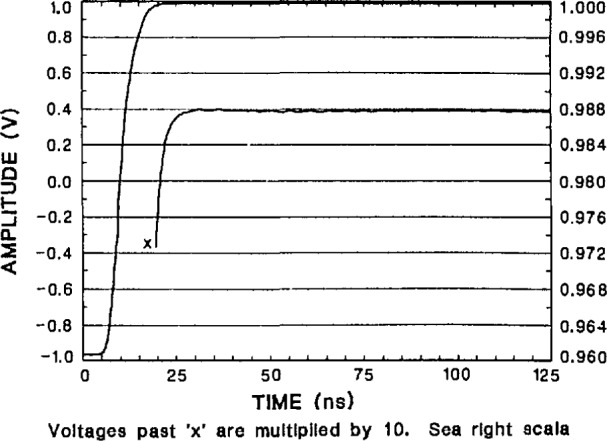
Response of the NIST SVT to a 2-V step from a slower, more accurate version of the NIST programmable step generator, showing settling to within ±0.02% of FSR in 20 ns.

**Figure 34:**
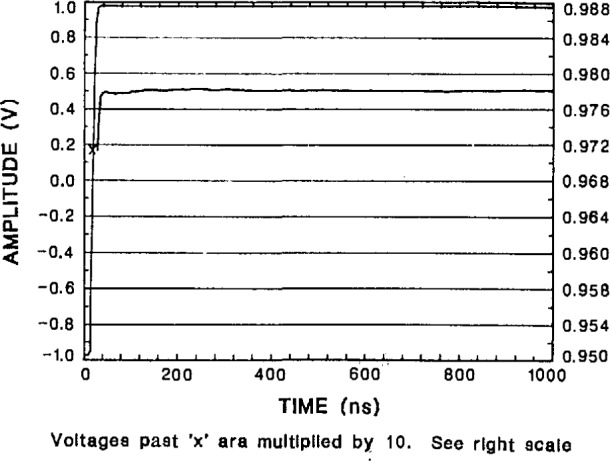
Longer term response of the NIST SVT to a step from the slower NIST programmable generator, showing abberations less then 0.4 mV (0.02% of FSR) out to 1 μs.

The transition duration (rise time) and settling-time performance of the NIST SVT is shown in figures 32, 33, and 34. The measurement of fast step-response performance at high accuracy is difficult because of the lack of reference waveform generators having the requisite characteristics. Some of the best reference waveform sources available for this work have been those developed by NIST [44,45,46]. The NIST programmable voltage step generator was used to obtain the short and long term settling response of the SVT shown in figures 32, 33, and 34. The step response data given in table 3 are derived from these plots.

**Figure 35:**
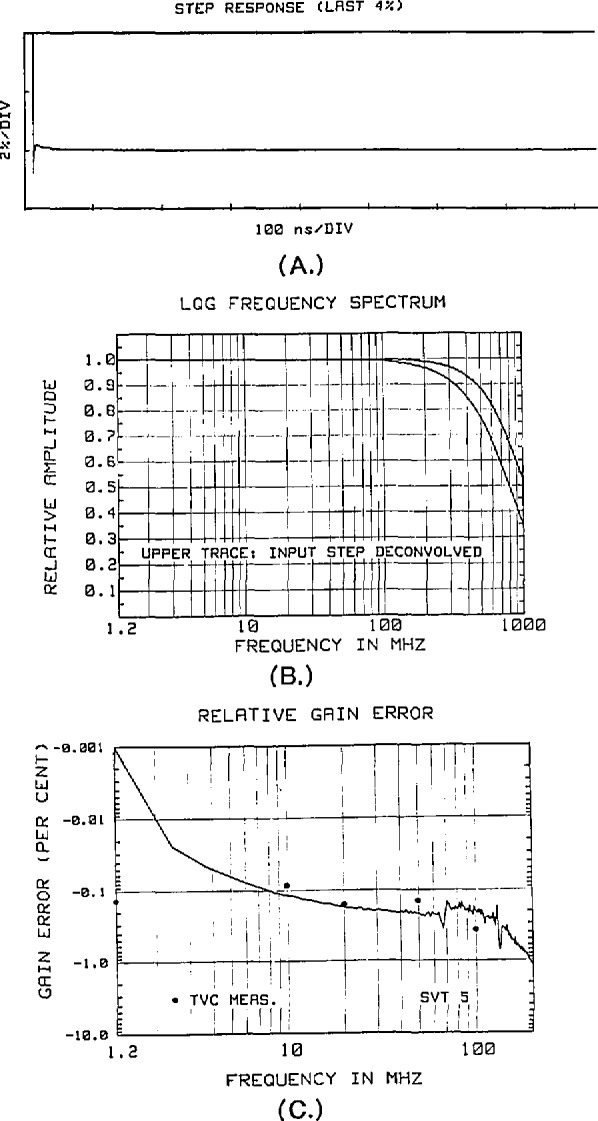
Gain corrections derived from measurements of the NIST SVT response to the output from a fast (~400 ps transition duration) step generator: (a) the last 4% of the step response, (b) the corresponding frequency response computed from the step response data, and (c) the gain error relative to the first spectral line at 1.2 MHz.

**Figure 38:**
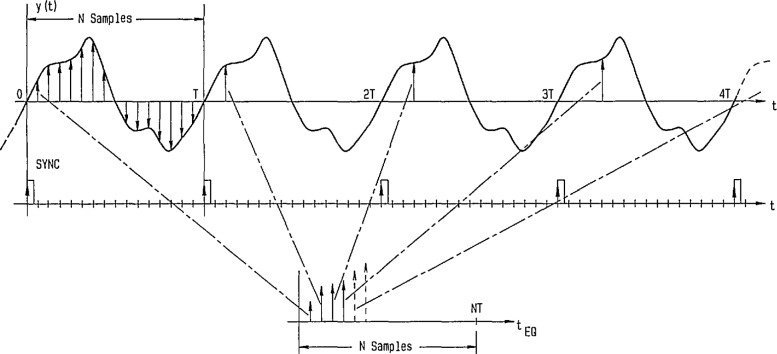
Illustration of a typical equivalent-time sampling process. Waveform samples are obtained at a rate *N* times slower than in real time sampling.

Accurate measurement of the frequency response of the NIST SVT is also difficult, since the direct measurement requires using sine-wave sources having high spectral purity and known amplitude uncertainty in the 1 to 1000 MHz range. Alternatively, indirect measurements based on time-domain data require fast risetime, fast settling step generators, preferably with transition durations of 300 ps or less [47]. The step generator used for these measurements is an improved, commercial version of the NIST Reference Flat Pulse Generator [44] with the manufacturer-specified rise time of ~400 ps. The step is sampled with an equivalent-time sampling rate sufficient to give negligible aliasing errors over the frequency range of interest. The discrete impulse response is then computed from the step-response data, and the frequency-magnitude spectrum is obtained by a Fourier transform of the impulse response. For one type of comparator that was used in the NIST SVT, figure 35 (a–c) shows: (a) the last 4% of the sampled input step, (b) the frequency response computed from this data, and (c) the gain error relative to the first spectral line at 1.2 MHz. The lower trace of figure 35 (b) is the frequency response of the composite step generator-SVT system. The upper trace is an estimate of the actual frequency response of the NIST SVT itself, obtained by deconvolving the equivalent response of the (nonideal) input step, estimated from rise time measurements of the input step and an assumed single pole model.

## 5. Conclusion

By the mid 1970s it was apparent that digital techniques would have significant advantages over analog methods for reducing the problems of offset, drift, and noise in making precise electrical measurements. Also, the proliferation of digital computers into laboratories and offices in the 1980s spurred the need to convert analog signals into digital form and vice versa. Consequently, there has been a corresponding requirement for better means to support the performance specifications of digital devices and associated instrumentation.

The NBS/NIST program in the research and development of new test methods and physical standards for this purpose has provided the improved capabilities described in this article. These digital-based standards and associated test methods also have been described in numerous journal and conference papers and talks. The work now provides the basis for new, improved NIST calibration services and special tests.

Future efforts will likely concentrate on similar approaches for supporting the devices and instruments used in automatic test systems, such as highspeed digitizers and arbitrary waveform generators. Characterizing the performance of electronic stimulus and measurement devices continues to be a challenge as the testing technology changes.

## Figures and Tables

**Figure 36:**
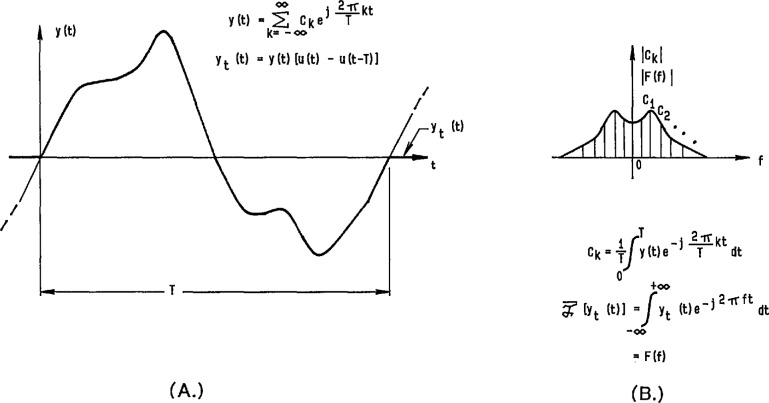
Time domain (a) and frequency domain (b) relationships for both a periodic and single occurrence arbitrary waveform.

**Figure 37:**
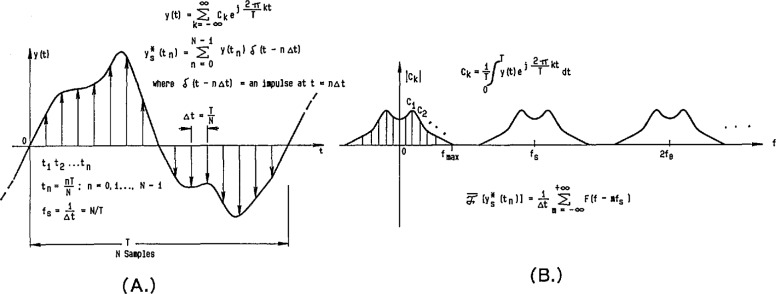
Time domain (a) and frequency domain (b) relationships for a sampled arbitrary waveform.

**Table 1:** Estimated maximum phase angle uncertainty (in mil-lidegrees)

	60 Hz	400 Hz	5 kHz	15 kHz	30 kHz	50 kHz
Auto-zero corr.^a^	±1	±2	±5	±6	±12	±20
Linearity	±2	±2	±3	±10	±15	±20
7:1 voltage ratio	±1	±2	±3	b	b	±40

a The uncertainty due to frequency adjustment is included.

b Data not available.

**Table 2:** Comparison of rms error and THD for unfiltered and filtered stepped sine waves

No. of steps per period	Unfiltered Reconstruction		Filtered Reconstruction	
	Rms error	THD	Rms error	THD
20	0	8.9%	−0.4%	0
200	0	0.9%	−0.004%	0

**Table 3:** Performance summary of the SVT

	dc	1	10	20	50	100	200	300	MHz
Linearity Error									
1 V (peak)	0.002	0.03	0.06	0.08	0.39	1.4	4.2	9	%
50 mV (peak)			0.06	0.12	0.20	0.2	0.7	1.3	%
Gain Error	0		0.004	0.02	0.30	0.7	−0.3	−4.5	%
	
Bandwidth (3 dB)				600					MHz
Step Response									
•Transition Duration (10–90 %)				600					ps
•Settling Time (−1 to +1 V step)									
0.1 %				4					ns
0.02 *%*				20					ns
•Long Term Settling Error									
(0–1 μs) −1 to +1 V step				±0.4					mV
Time Base Linearity Error									
100 ns epoch				<2					ps rms
Time Base Phase Noise, > 1 Hz				<10					ps peak

## Footnotes

1 Uncertainties quoted in this paper are taken from the references given and are not uniformly defined.

## 6. Appendix

### 6.1 Relationship Between *y_z_*(*t*) and Its Fourier Series Line Spectrum

In the notation of figure 8(a), *f*_s_=*N/T* is the sampling frequency, and the sampling times are *t_n_*=*nT/N*=*n/f*_s_, *n*=0, 1,…,*N*−1. Then, over the time interval 0⩽*t*⩽*T*, the stepped waveform *y_z_*(*t*) can be written as

$$y_{zN}\left(t\right)=\sum_{n=0}^{N-1}y\left(t_{n}\right)\left[u\left(t-t_{n}\right)-u\left(t-t_{n+1}\right)\right],$$ (1A)

where *u*(*t*) is the usual unit step function, conveniently defined as a discontinuous step that occurs when its argument is zero, i.e.,

$$\begin{array}{rr} u\left(t\right)=0, & t<0\\ =1, & t\geq 0. \end{array}$$

Hence, a rectangular pulse between *t_n_* and *t_n_*_+1_ is readily described by the time difference of two such step functions, and the stepped waveform is created by indexing *n* over the *N* sample points on the waveform. Extending *y_zN_*(*t*) periodically for all *t*, one obtains the periodic function *y_z_*(*t*), which can be written as a Fourier series of exponential terms,

$$y_{z}\left(t\right)=\sum_{k=-\infty }^{+\infty }C_{k}\cdot e^{j2\pi kt/T},$$ (2A)

or, as indicated in the line spectrum of figure 8(b), as a trigonometric Fourier series,

$$y_{z}\left(t\right)=A_{0}/2+\sum_{k=1}^{+\infty }A_{k}\cdot \cos\left(2\pi f_{k}t+\theta_{k}\right),$$

where

$$\theta_{k}=-\tan^{-1}\left[Im\left(C_{k}\right)/Re\left(C_{k}\right)\right],\;f_{k}=k/T,$$

and *A_k_*(*f*) vs. frequency is the plot of the line spectrum.

Given the *C_k_* coefficients of eq (2A)*A_k_* can be found from

$$A_{k}=2{\left(C_{k}\cdot C_{-k}\right)}^{1/2}.$$ (3A)

It is known that the Fourier transform of a continuous periodic function such as *y_z_*(*t*),

$$F\left(y_{z}\left(t\right)\right)=\int_{-\infty }^{+\infty }y_{z}\left(t\right)\cdot e^{-j2\pi ft}dt,$$ (4A)

is a sum of equally spaced impulses in the frequency domain, i.e.,

$$F\left(y_{z}\left(t\right)\right)=F\left(f\right)=2\pi \sum_{k=-\infty }^{+\infty }C_{k}\cdot \delta \left(f-k/T\right),$$

where the *C_k_* (coefficients of the exponential Fourier series) are related to the truncated function *y_zN_*(*t*) through the formula

$$C_{k}=1/T\int_{0}^{T}y_{zN}\left(t\right)\cdot e^{-j2\pi kt/T}dt,$$ (5A)

and δ(*f*) is the usual Dirac unit impulse function, conveniently defined as an infinite amplitude spike (with an area of one) that occurs when its argument is zero, i.e.,

$$\begin{array}{l} \delta (f)=\infty ,\\ \;=0 \end{array}\;\begin{array}{l} f=0\\ \mathrm{elsewhere}, \end{array}$$

and

$$\int_{-\infty }^{+\infty }W\delta \left(f\right)df=W,$$

where *W* is the area under (or “weight” of) the impulse.

[See reference [19] (Papoulis, Ch. 3); in particular, the integral in eq (4A) has to be interpreted in the sense of distributions (Papoulis, Appendix I)].Since

$$\begin{array}{l} 1/T\int_{0}^{T}\left[u\left(t-t_{n}\right)-u\left(t-t_{n+1}\right)\right]e^{-j2\pi kt/T}dt\\ \;=e^{-j2\pi kn/N}\cdot \frac{1-e^{-j2\pi k/N}}{j2\pi k/T}, \end{array}$$

then eq (5A) can be evaluated as

$$C_{k}=b/T\sum_{n=0}^{N-1}\sin\left(2\pi n/N\right)\cdot e^{-j2\pi kn/N}\cdot \frac{1-e^{-j2\pi k/N}}{j2\pi k/T}.$$ (6A)

The summation over *N* terms of the sin (2*πn/N).e^−j2πkn/N^* combination can be computed by means of the formula for finite geometric sums since

$$\begin{array}{l} \sin\left(2\pi n/N\right)\cdot e^{-j2\pi kn/N}\\ \;=\frac{{\left\{e^{j2\pi \left(1-k\right)/N}\right\}}^{n}-{\left\{e^{-j2\pi \left(1+k\right)/N}\right\}}^{n}}{2j}. \end{array}$$ (7A)

If the factor 1−*k* or 1*+k* in the exponents is not a multiple of *N*, the corresponding summation is zero; hence, *C_k_*=0 if neither 1−*k* nor 1*+k* is a multiple of *N*. If 1−*k* is a multiple of *N* and, hence, 1+*k* is not, then the exponentials involving 1−*k* are equal to 1 (and sum up to *N*), and those involving 1+*k* sum up to zero. Thus, in this case

$$C_{k}=b\left(N/2j\right)\cdot \frac{1-e^{-j2\pi k/N}}{j2\pi k}.$$ (8A)

Similarly, if 1+*k* is a multiple of *N*,

$$C_{k}=-b\left(N/2j\right)\cdot \frac{1-e^{-j2\pi k/N}}{j2\pi k}.$$ (9A)

Equations (8A) and (9A) are valid for both positive and negative *k* values. To compute *A_k_* from eq (3A), either *C_k_* or C_−_*_k_* must be expressed by eq (8A) and the other coefficient by eq (9A). Thus,

$$\begin{array}{l} A_{k}=2\left\{b^{2}N^{2}/\left(16\pi^{2}k^{2}\right)\cdot \left(1-e^{j2\pi k/N}\right)\left(1-e^{-j2\pi k/N}\right)\right\}{\;}^{1/2},\\ \;=bN/2\pi k\cdot {\left\{2-2\cos\left(2\pi k/N\right)\right\}}^{1/2},\\ \;=bN/\pi k\cdot \sin\left(\pi k/N\right). \end{array}$$ (10A)

Since *f*_s_=*N*/*T* and *f*_k_=*k*/*T*,

$$k/N=\frac{k/T}{N/T}=f_{k}/f_{s}.$$ (11A)

Therefore,

$$\left|A_{k}\right|=b\left|\frac{\sin\left(\pi f_{k}/f_{s}\right)}{\pi f_{k}/f_{s}}\right|,$$ (12A)

which is shown plotted in figure 8(b). QED.

### 6.2 Some Basics on Sampling Theory

Figure 36 (a) shows a periodic waveform of arbitrary shape in the time domain that can be described by a Fourier series (shown expressed as a series of exponential terms rather than the trigonometric series). Each term is one of a pair of complex conjugates whose coefficient, *C_k_*, is the amplitude of the corresponding frequency in the line spectrum of figure 36 (b). If this were a single arbitrary pulse, its frequency domain representation would be found by taking the Fourier transform:

$$\begin{array}{l} F\left[y_{t}\left(t\right)\right]=\int_{-\infty }^{+\infty }y_{t}\left(t\right)e^{-j2\pi ft}dt\\ \;=F\left(f\right), \end{array}$$ (13A)

where

*y_t_*(*t*) is the transient arbitrary pulse, and
*F*[ ] is the frequency dependent Fourier transform.

In this case, the frequency representation is a continuous function of frequency rather than a discrete line spectrum.

When synchronously sampling this waveform, as shown in figure 37 (a), the sample set mathematically can be given a discrete time domain representation *y*_s_*(*t_n_*) that is a series of impulses at the times *t_n_*, whose areas (or “weights”) are equal to *y*(*t_n_*):

$$y{*}_{s}\left(t_{n}\right)=\sum_{n=0}^{N-1}y\left(t_{n}\right)\delta \left(t-n\Delta t\right),$$ (14A)

where

Δ*t* = *T/N* is the sampling time interval,
*T* is the period or record length of the sample set,
*N* is the total (integral) number of samples, and
Δ(*t* − *n* Δ*t*) is a unit impulse at *t* = *n* Δ*t*.

The sampled time function has a corresponding discrete Fourier transform (DFT), which can be shown to be the regular Fourier transform shifted along the frequency scale at infinite multiples of the sampling frequency [33], where *f*_s_=1*/Δt=N/T* [see fig. 37 (b)]. Obviously, to prevent overlapping spectra (or aliasing) requires that

$$f_{s}⩾2f_{\max}.$$ (15A)

For periodic waveforms, nonoverlapping requires that

N⩾2k+1. (16A)

These are the usual forms for the well-known Nyquist frequency criterion that comes from communication theory [34].

In order to satisfy the Nyquist frequency criterion when the highest frequency in a periodic signal becomes comparable to the available maximum sampling frequency, waveform sampling is done in “equivalent-time” rather than in real time. Figure 38 is an illustration of equivalent-time sampling where *N* samples of the waveform are taken over equivalent time *NT*, rather than taking *N* samples in real time *T*. Each sample is obtained by incrementing one additional sampling interval (relative to the sync pulse) in each successive period of the signal. Multiple sets of *N* samples may be obtained, and averaging performed on corresponding sample points, in order to reduce the effects of noise and jitter. This method works even for signals with frequency components above the sampling frequency, as long as the effective sampling frequency (reciprocal of the equivalent-time sampling interval) satisfies the Nyquist criterion. Equivalent-time sampling requires periodic signals that are cycle-to-cycle repeatable (except for noise and jitter limitations). Also, as with real time sampling, the time base must be linear over the time interval *T*, and stable over the record length, as well as accurate.
